# A Short Review on Copper Calcium Titanate (CCTO) Electroceramic: Synthesis, Dielectric Properties, Film Deposition, and Sensing Application

**DOI:** 10.1007/s40820-016-0089-1

**Published:** 2016-03-30

**Authors:** Mohsen Ahmadipour, Mohd Fadzil Ain, Zainal Arifin Ahmad

**Affiliations:** 1 Structural Materials Niche Area, School of Materials and Mineral Resources Engineering, Universiti Sains Malaysia, Engineering Campus, 14300 Nibong Tebal, Penang Malaysia; 2 School of Electrical and Electronic Engineering, Universiti Sains Malaysia Engineering Campus, 14300 Nibong Tebal, Penang Malaysia

**Keywords:** CCTO, Chemical synthesis, Dielectric permittivity, Loss factor, Deposition, Sensitivity

## Abstract

Electroceramic calcium copper titanates (CaCu_3_Ti_4_O_12_, CCTO), with high dielectric permittivities (*ε*) of approximately 10^5^ and 10^4^, respectively, for single crystal and bulk materials, are produced for a number of well-established and emerging applications such as resonator, capacitor, and sensor. These applications take advantage of the unique properties achieved through the structure and properties of CCTO. This review comprehensively focuses on the primary processing routes, effect of impurity, dielectric permittivity, and deposition technique used for the processing of electroceramics along with their chemical composition and micro and nanostructures. Emphasis is given to versatile and basic approaches that allow one to control the microstructural features that ultimately determine the properties of the CCTO ceramic. Despite the intensive research in this area, none of the studies available in the literature provides all the possible relevant information about CCTO fabrication, structure, the factors influencing its dielectric properties, CCTO immobilization, and sensing applications.

## Introduction

Calcium copper titanate (CCTO) has the chemical formula of CaCu_3_Ti_4_O_12_, a novel electroceramic material with high dielectric permittivity (*ε*), of approximately 100,000 for single crystal and 10,000 for bulk material at room temperature. In addition, CCTO shows moderate dielectric loss (tan *δ* ~ 0.15) at a broad frequency region (up to 10^6^ Hz), high positive temperature coefficient of resonant frequency (*τ*_f_ ~ +9.13 ppm K^−1^), and phase transition stability against temperatures of a wide range (100–400 K). In 2000, Subramanian et al. [1] discovered that CCTO belongs to the family of ACu_3_Ti_4_O_12_ (A = Ca, Sr, Ba, Bi_2/3_, Y_2/3_, La_2/3_)-type oxide of pseudo-cubic perovskite-related structure (space group: Im^3^) with a lattice parameter of 7.391 Å. The huge value of *ε* remaining constant over a wide range of temperatures from 100 to 400 K for CCTO allows for its use in wide potential applications [2–4].

The advancement of technology requires a material with giant *ɛ* value to reduce the size of electronic components, whereas the effective performance of these electronic components requires substantially low tan *δ*. For this reason, a great number of theoretical and experimental researches have been carried out to reveal the nature and the origin of the giant *ɛ* value of CCTO ceramics. Various processing routes (chemical and physical methods) of CCTO were adopted such as solid-state reaction, wet-chemistry route, sol–gel, solution combustion synthesis, sonochemical-assisted route, and co-precipitation [5–10]. The solid-state reaction is a common method for the synthesis of CCTO from CaCO_3_, CuO, and TiO_2_ at high temperatures accompanied by drawbacks like heterogeneity of precursor materials and long reaction time [11–14]. A few other drawbacks are secondary phases which appear during the synthesis because of limited atomic diffusion through micrometer-sized grains. It is well known that the electric properties can be remarkably enhanced when ceramic has a homogeneous microstructure. Other methods are available to improve CCTO characteristics, but not without some drawbacks.

The study of CCTO has been attracting interest due to its molecular structure and wide applications. For instance, it has been applied in capacitors, antennas, microwave devices, and sensors. CCTO for sensor applications shows benefit from the point of view of its polycrystalline porous nanostructures. One of the most significant properties of CCTO sensor is to detect and monitor gases and toxic species without decomposition or change in their structural arrangements. Researches have been carried out on CCTO to enhance its sensitivity and selectivity on gas sensing, chemical and bio-sensors. However, little work has been published on some aspects of CCTO’s sensing applications, and this still remains as a potential research area. The CCTO gas sensor is considered non-ohmic device because electric properties are greatly dominated by grain–boundary interface states [15]. These non-ohmic ceramic devices are also known as “metal-oxide” varistors (variable resistors) applications of which are technologically important because of their electric characteristics that enable them to be used as solid-state switches with large-energy-handling capabilities. The varistors are also know as voltage-dependent resistors because they show a highly nonlinear current–voltage (*I*–*V*) characteristic. The first voltage-dependent resistors polycrystalline ceramics were developed around early the 1930s by the Bell System and consisted of partially sintered compacts of SiC. A voltage-dependent resistor-based system with very superior performance based on CCTO composition was announced in 2005 by Il-Doo Kim [16] although parallel developments were reported in Brazil in the early part of 2008 [17]. This research revealed a mechanism responsible for remarkable electron transportation (n-type or p-type conductivity) which depends on the synthesis methods and experimental conditions [18, 19].

This study reviews the principal methods for the fabrication of CCTO, and the determination of its structure, factor influence on dielectric properties, the immobilization, and sensing applications. Apart from the limited studies by researchers on CCTO electroceramic as a gas sensor, there are no comprehensive reviews on its sensing applications, while even the few available only focus on the dielectric properties of bulk ceramics and dense films.

## Synthesis Methods of CCTO

Different synthesis methods have been adopted by various researchers to obtain CCTO electroceramic in order to tailor its dielectric properties.

### Solid-State Reaction Method

**Fig. 1:**
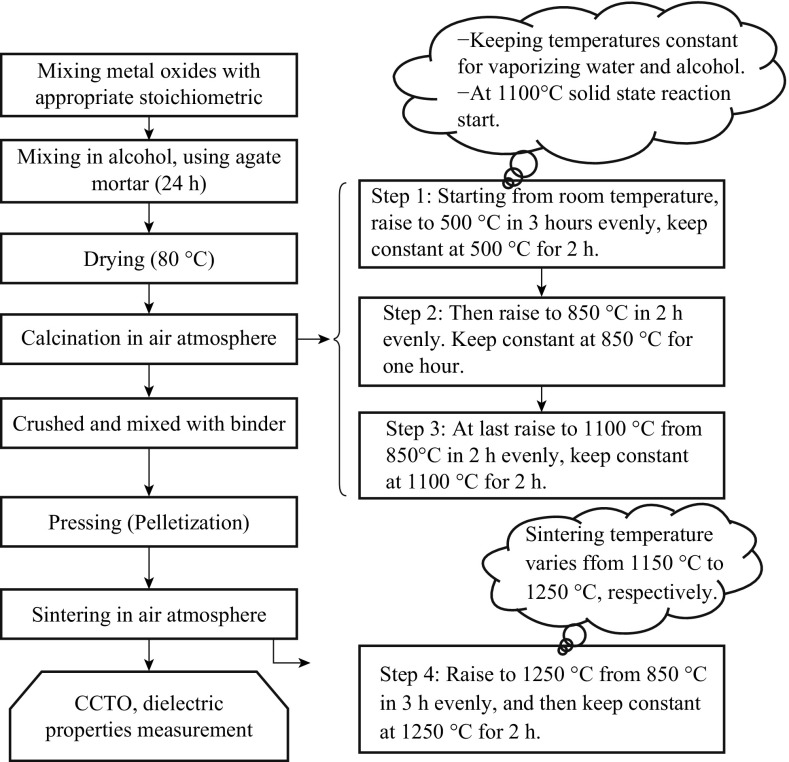
Flow chart for the synthesis of pure CCTO by solid-state route

CCTO is generally synthesized by a conventional solid-state method or dry route [20, 21]. The method normally uses stoichiometric amounts of the common precursors, i.e., CaCO_3_, CuO, and TiO_2_ for being mixed with suitable liquid (acetone or ethanol), using ball mill. To remove volatile impurities, the fine powder is calcined at 930 °C for a designated period of time (12 h). An appropriate amount (1 cc mL^−1^) of a suitable binder (PVA) is added to the powder and mixed uniformly before being compressed to form a pellet shape. The resulting product is first heated slowly (at a heating rate 5 °C min^−1^) to a particular temperature (1040 °C) to burn off the binder. The mixture is then maintained at this temperature for 10 h for annealing. The sample is later cooled under a controlled rate of cooling (cooling rate 5 °C min^−1^). A systematic flow chart of a solid-state reaction method is shown in Fig. 1.

The chemical reaction during the conventional solid-state reaction method for the synthesis of CCTO is shown in Eq. 1:

1 $${\text{CaCO}}_{{\begin{array}{*{20}c} {3 } \\ \end{array} }} + 3{\text{CuO}} + 4{\text{TiO}}_{2} \to {\text{CaCu}}_{3} {\text{Ti}}_{4} {\text{O}}_{12} + {\text{CO}}_{2} \uparrow$$

Shao et al. [22] prepared CaCu_3_Ti_4_O_12_ ceramics by the conventional solid-state reaction method under various sintering temperatures from 1000 to 1120 °C at intervals of 10 °C. It was reported that the morphologies changed significantly with the sintering temperature. Ceramic specimens prepared by this method had a good polycrystalline structure in spite of the different microstructures. The *ɛ* value was found to increase with the increasing sintering temperature and have a close relation with the polycrystalline microstructure and particularly the grain size.

However, solid-state route method suffers the disadvantages of inhomogeneity, and the need for repetitive grinding, the need for firing at high temperatures, and a prolonged reaction time.

### Wet-Chemistry Method

**Fig. 2:**
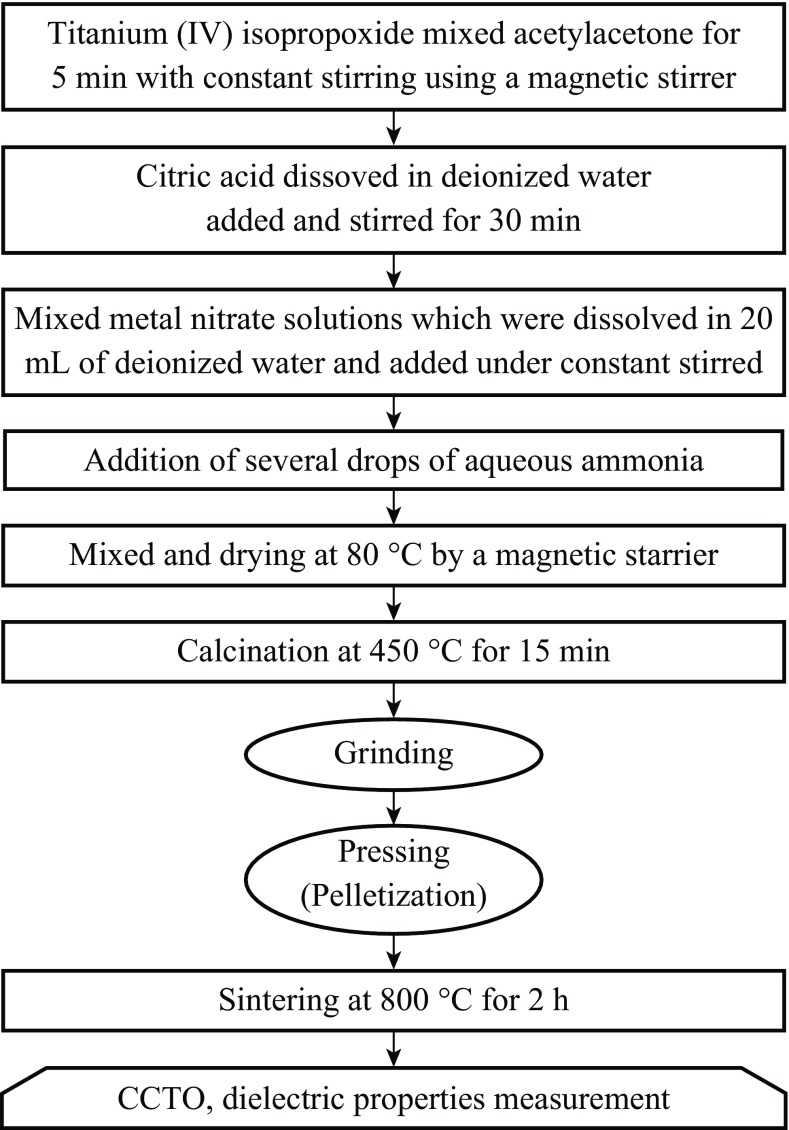
Flow chart for the synthesis of materials by a wet-chemistry method

Wet-chemistry method is a type of combustion synthesis technique that is based on redox reaction between a fuel and an oxidant in a precursor solution. In general, citric acid, urea, ethylene glycol, etc. are used as a fuel, while nitrates of different metals are used as oxidants. The chelating agents like EDTA, acetic acid, etc. can form complex with metal ions in the precursor solution and act as a fuel. This complex in the dehydration process produces a viscous gel which can further undergo self-ignition along with the evolution of huge amount of gases. In the combustion method, the calcium/copper/titanium/citrate aqueous solution becomes a viscous, transparent blue gel after the evaporation of water. After heat treatment, all organic materials burned out, and a voluminous porous black sample is left behind. This leads to the remnant of fine phase pure powder. The systematic flow chart of the wet-chemistry method is depicted in Fig. 2.

Liu et al. [23] reported the synthesis of fine CCTO powder using the wet-chemistry method at relatively low temperatures and with a shorter reaction time. The pure-phase sample was obtained at 800 °C after sintering for 5 h, and the grain-sized pellet sample was sintered at 1030 °C for 4 h. The samples had a homogeneous distribution in the range of 0.4–1.5 μm. This method started with the mixing a homogeneous liquid solution with cation ingredients, in stoichiometric ratio at the atomic scale. Therefore, pure samples at nanoscale could theoretically be obtained at lower temperature and a shorter reaction time than that obtained by solid-state reactions.

### Sol–Gel Method

**Fig. 3:**
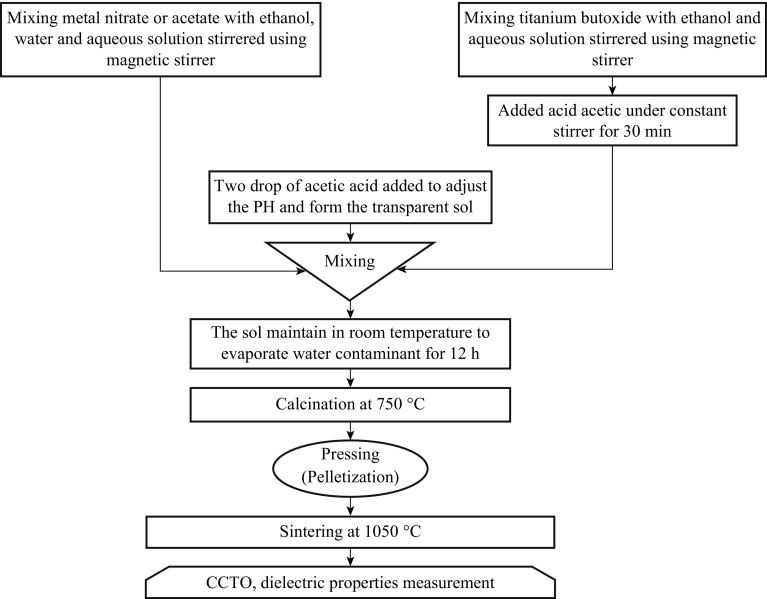
Flow chart for the synthesis of materials by a sol–gel method

This method is also known as the Pechini, or liquid mix process and widely used in the fields of material science and ceramic engineering. The sol–gel process is also known as chemical solution deposition. In 1967, Maggio P. Pechini developed a sol–gel method for lead and alkaline earth titanates and niobates, materials that do not have favorable hydrolysis equilibria. Such methods are used primarily for the fabrication of materials (typically a metal oxide) starting from a chemical solution (or sol) that acts as the precursor for an integrated network (or gel) of either discrete particles or network polymers. Typical precursors are metal alkoxides and metal chlorides, which undergo various forms of hydrolysis and poly condensation reactions. Factors that need to be considered in a sol–gel process are solvents, temperature, precursors, catalysts, pH, additives, and mechanical agitation. These factors can influence the kinetics, growth reactions, hydrolysis and condensation reactions. The solvent influences the kinetics and conformation of the precursors, while pH affects the hydrolysis and condensation reactions. Acidic conditions favor hydrolysis, which means that fully or nearly fully hydrolyzed species are formed before condensation begins. Under acidic conditions, there is a low crosslink density, which yields a denser final product when the gel collapses. These reactions are carried out at room temperature, and further heat treatments need to be conducted for obtaining the final crystalline state. Sol–gel routes can be used to prepare pure, stoichiometric, dense, particles of CCTO [24–26]. The flow chart of synthesis of CCTO is shown in Fig. 3.

Liu et al. [27] have synthesized CCTO using the giant ɛ material by a sol–gel method using nitrate and alkoxide precursors. The *ɛ* of CCTO was found to be three times smaller than that synthesized by other low-temperature methods including solid-state reaction method. Their results were explained by internal barrier layer capacitor (IBLC) model of Schottky barriers at grain boundaries between semiconducting grains. A sol–gel process showed considerable advantages, including excellent chemical stoichiometry, compositional homogeneity, and lower crystallization temperature due to the mixing of liquid precursors at the molecular level [28, 29] compared with other techniques. Ion-diffusing displacement is shortened in a sol–gel process. The pure phases of the powders were obtained upon calcination at 900 °C for 1 h. The *ɛ* of CCTO ceramics was found to be 35,000 at 1 kHz in sintered samples at 1060 °C for 48 h.

### Combustion Synthesis Technique

Combustion synthesis in solid-state chemistry is also known as metathesis reaction or self-propagating high-temperature synthesis (SHS). By this method, homogeneous nanopowder, and multi-component single-phase material can be produced. The SHS is one of the effective and economic methods [30] and has emerged as an important technique for the synthesis and the processing of advanced ceramics, catalysts, composites, alloys, intermetallics, and nanomaterials [31]. The method exploits self-sustaining solid flame-combustion reaction for the internal development for a limited short period [32]. The combustion synthesis was first studied by Alexander Merzhavov in 1970s [33] and was reviewed by Gillan and Kaner [34]. A typical reaction occurred between a metal halide and an alkali or alkaline earth main group compounds.

The emphasis in the ceramic synthesis is the need for high reaction temperatures in order to achieve practically usable coefficients of diffusion for the atoms in the reacting solids. Mechanical alloying offers an alternative to high reaction temperatures (i.e., high-pressure pulses), which themselves can be a source for local rapid heating. In the combustion synthesis, high temperatures again take center stage. Consequently, the high temperature is achieved not by placing the sample in a furnace, but by means of the heat generated by exothermic chemical reaction that sustains the high temperature for a duration of around 30–45 s to form the desired end-product of the reaction. Thus, the heating is spontaneous, releasing a large amount of gases during the combustion process with the consequent formation of a nanosized, porous, and foamy product. The mechanism of the combustion reaction is quite complex. The parameters that influence the reaction kinetics and mechanisms include the type of fuel, the fuel-to-oxidizer ratio (*Ψ*), the use of excess amount of oxidizer, the ignition temperature, and water content of the precursor mixture. It is well recognized that the fuel is an important component for the preparation of oxides by combustion synthesis. If the fuel-to-oxidizer ratio is unity, the energy released by the combustion is maximized. Fuel-to-oxidizer ratio is calculated and expressed as shown in Eq. 2:

2 $$\psi = \frac{\text{Oxidizing and reducing elements in fuel}}{\text{ All oxidizing and reducing elements in oxidizer}}$$

The ratio is crucial and effective in this method [35, 36]. It can change the properties of the nanomaterial as the reaction temperature is dependent on fuel-to-oxidizer ratio. In general, citric acid, urea, or ethylene glycol is used as a fuel, and nitrates of different metals are used as oxidants.

**Fig. 4:**
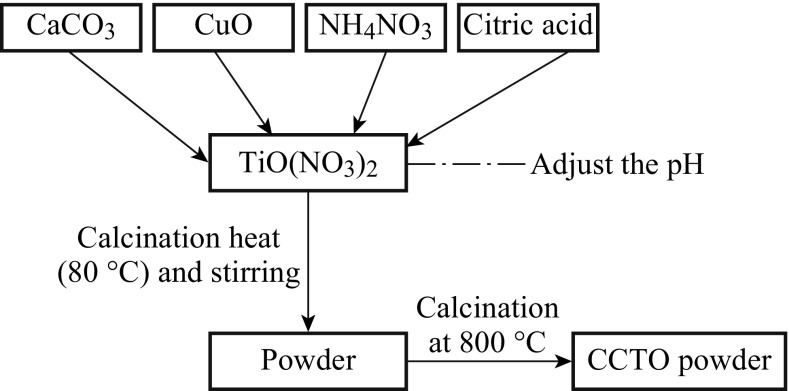
Flow chart of CCTO powder synthesis by a solution combustion method [37]

Patra et al. [37] have completely synthesized the nanopowder CCTO by citrate–nitrate gel combustion technique followed by calcination in the temperature range of 700–800 °C. The average crystalline size of synthesized powders was 66 nm, and the particle size ranged between 189 and 300 nm. Sintering of the powders was conducted in air at 1000 °C for 4–6 h. The X-ray diffraction (XRD) study revealed that CCTO powder calcined in 700 °C had some extra peaks that were unable to be identified at 800 °C, where XRD showed pure CCTO without any impurity. Scanning electronic microscopy (SEM) images of the sintered CCTO ceramics showed submicron grain size distribution of the sample in 1000 °C (4 h)^−1^ and an illustrated matrix consisting of large grains, wherein the small grains were embedded between the large grains at 1000 °C (6 h)^−1^. The samples exhibit very high dielectric permittivities of 6800 and 23,200, and dielectric losses of 0.21 and 0.61 for 1000 °C (4 h)^−1^ and 1000 °C (6 h)^−1^ at 1 kHz, respectively. This method was found very attractive due to its relatively greater effectiveness and economy in operational cost for obtaining a homogeneous and fine powder precursor. Figure 4 shows the combustion reaction flow chart.

### Sonochemical-Assisted Process

Sonochemical originates from the extreme transient conditions induced by ultrasound which produces unique hot spots that can withstand temperatures and pressures of, respectively, above even 5000 K and 1000 atmospheres, with the heating and cooling rates being in excess of 1010 K s^−1^. The speed of sound in a typical liquid is 1000–1500 m s^−1^, and the ultrasonic wavelengths will vary from roughly 10 cm down to 100 µm over a diminishing frequency range of 20–15 MHz, which is much larger than the molecular-size scale. The chemical and physical effects of ultrasound, therefore, arise not from a direct interaction between chemical species and sound waves, rather from the physical phenomenon of acoustic cavitation [38] that involves (a) formation, (b) developing, and (c) the implosive collapse of the microcavities. The acoustic waves crossing the liquids are generating a cavity phenomenon. When sound waves with sufficient amplitude propagate through a liquid, the liquid is under dynamic tensile stress, and the density changes with alternating expansive and compressive waves. Bubbles are generated from pre-existing impurities (e.g., gas-filled crevices in dust motes) and oscillate with the applied sound field. Bubbles can grow through a slow pumping of gas from the bulk liquid into the oscillating bubble (rectified diffusion). Bubbles at a critical size (usually tens of micrometers) can couple strongly in response to the extreme transient conditions produced during acoustic cavitation. This allows for the formation of unique materials and also permits syntheses on the benchtop in a room-temperature liquid which would otherwise require high temperatures, high pressures, or long reaction times. When a liquid is irradiated by high-intensity ultrasound, high-energy chemical reactions occur. Sonochemistry was employed in the synthesis of materials from volatile or nonvolatile precursors, but generally involving different mechanisms. Sonochemical activation of a starting material led to a precipitation reaction that improves the homogeneity of the final product.

**Fig. 5:**
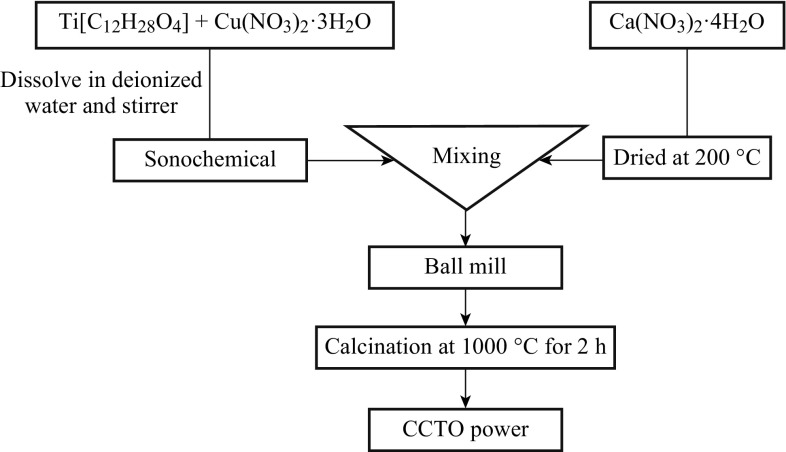
Flow chart of CCTO powder synthesis by sonochemical-assisted process [39]

Wongpisutpaisan et al. [39] synthesized the giant dielectric-constant material CCTO by aonochemical-assisted process containing stoichiometric amounts of the metal nitrate, at a shorter reaction time than that of a conventional solid-state reaction. Synthesis from a solution affords intimate and homogeneous mixing of the metal ions at the atomic scale. Thus, this leads to the reduction of the diffusion path length. However, a short diffusion length reduces reaction time. The diameter of the multiphase CCTO powders was 500 nm as obtained by SEM, and the average crystalline size calculated using the Scherrer’s equation from the full-width-at-half-maximum (FWHM) of the strongest diffraction peak (220) was ∼75 nm. Wongpisutpaisan et al. [39] observed that as the frequency increased over 1 kHz, the *ɛ* rapidly decreased. In addition, the higher *ɛ* values at lower frequency could be related to the possibility of the presence of interfacial polarization. The systematic flow chart is illustrated in Fig. 5.

### Co-precipitation Method

Co-precipitation reactions are among the oldest techniques for the synthesis of nanomaterials. The reaction precipitate substances are normally soluble under the reaction conditions. There are three main mechanisms of co-precipitation: inclusion, occlusion, and adsorption mechanisms [40]. An inclusion occurs when the impurity occupies a lattice site in the crystal structure of the carrier. The inclusion of impurities results in a crystallographic defect that occurs when the ionic radius and charges of the impurity are similar to those of the carrier. An adsorbate is an impurity that is weakly bound (adsorbed) to the surface of the precipitate. An occlusion occurs when an adsorbed impurity gets physically trapped inside the crystal as it grows.

**Fig. 6:**
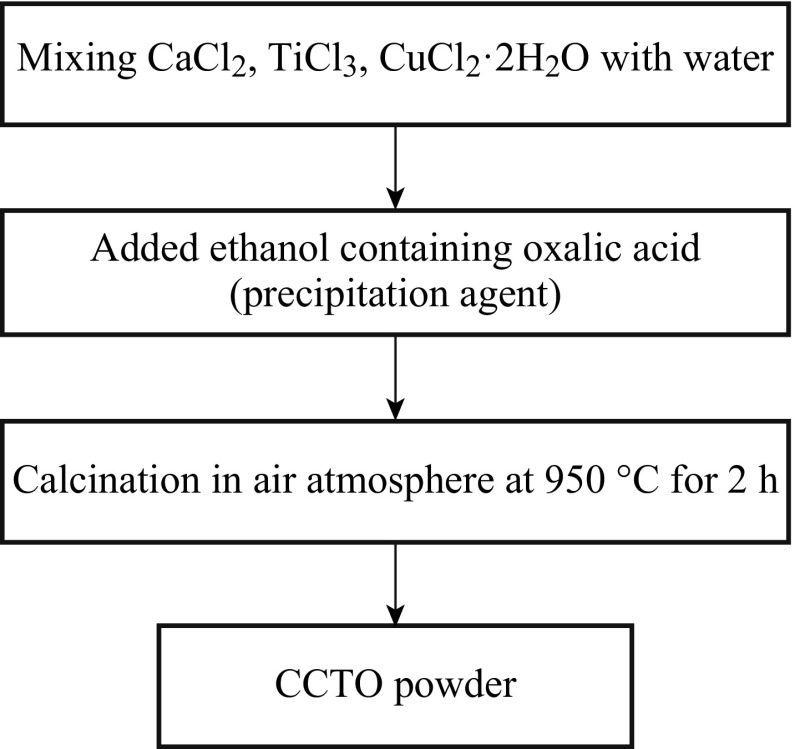
The flow chart of CCTO powder synthesis procedure by the co-precipitation method

**Fig. 7:**
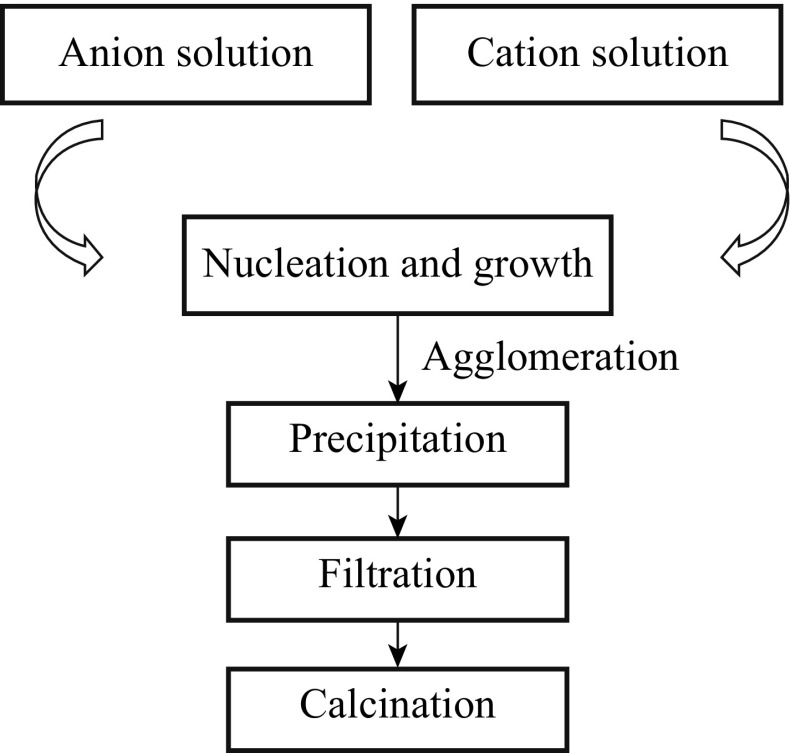
Preparation of CCTO by the co-precipitation method

Barbier et al. [10] synthesized CCTO powders by a soft chemistry method (co-precipitation method). The sintered pellets showed a high room temperature *ɛ*_r_ (∼1.4 × 10^5^) and relatively small tan *δ* (∼0.16) at 1 kHz. The study suggested that the high dielectric permittivity observed in the material were not related to an interface but, related to mechanism due to the type of the capacitor (an internal barrier layer capacitor (IBLC)) used. The samples prepared from the powder were found to exhibit a bimodal grain size distribution, with small grains of about 20 μm and large grains of size ranging from 50 to 200 μm. Further, they found that the nature of the electrode contact had no influence on the *ɛ*_r_ and the amounts of loss of CCTO pellets. The *ɛ*_r_ strongly depends on the sample diameter, but, the tan *δ* remains constant regardless of diameter. The systematic flow chart of preparation of CCTO powder by a co-precipitation method is shown in Figs. 6 and 7. Table 1 shows the comparison of CCTO powder-preparation methods in terms of particle size. Similarly, the advantages and disadvantages of the various production methods are explained.

**Table 1:** Various routes for the CCTO synthesis

Method	Material	Particle size	Advantage	Disadvantage	References
Solid-state method	CaCO_3_, TiO_2_, CuO	3 μm	Produced large amounts Easy to carry out the Synthetic procedure Starting materials readily available	Requires relatively long reaction High-temperature condition Secondary phases appear	[32]
Wet-chemistry method	Ca(NO_3_)_2_·4H_2_O, Cu(NO_3_)_2_·2.5H_2_O, Ti[OCH(CH_3_)_2_]_4_, citric acid, acetylacetone, ethylene glycol	4–15 μm	Low cost, reliability High throughput, Excellent selectivity	Very hard to control critical feature dimension Hazardous and difficult to handle Toxic Fume	[54]
Sol–gel method	Ca(NO_3_)_2_·4H_2_O, Cu(NO_3_)_2_·4H_2_O, CH_3_OCH_2_CH_2_OH,Ti Sol	50 nm	Lower temperatures for processing Can be used to make nanostructured powders, films, fibers	Starting materials are very expensive	[58]
Sol–gel method	Ti(OC_4_H_9_)_4_, Ca(OOCCH_3_)_2_·H_2_O Cu(OOCCH_3_)_2_·H_2_O	260 nm			[59]
Combustion synthesis method	TiO(NO_3_)_2_, CaCO_3_, citric acid, NH_4_NO_3_	189–300 nm	Low-cost and low-temperature process, rapid process Better control of stoichiometry Possibility of multicomponent oxides with single phase and high surface area Exothermic reaction makes product almost instantaneously	Contamination due to carbonaceous residue, particle agglomeration, poor control on particle morphology	[62]
Sonochemical-assisted method	Ca(NO_3_)_2_·4H_2_O Cu(NO_3_)_2_·3H_2_O Ti [C_12_H_28_O_4_]	75 nm	Nonhazardous, rapid reaction rate, produces very small metal particles	Can be combined with oxidation or advanced	[52]
Co-precipitation method	CaCl_2_, TiCl_3_ CuCl_2_·2H_2_O Ethanol, oxalic acid used	–	Useful tool for sampling, purifying solutions, and cleaning up environmental hazards Homogeneous mixing of reactant precipitates reduces the reaction temperature	It is not suitable for the preparation of high pure, accurate stoichiometric phase It does not work well, if the reactants have very different solubility as well as different precipitate rates	[37]

## Structure and Dielectric Properties

**Fig. 8:**
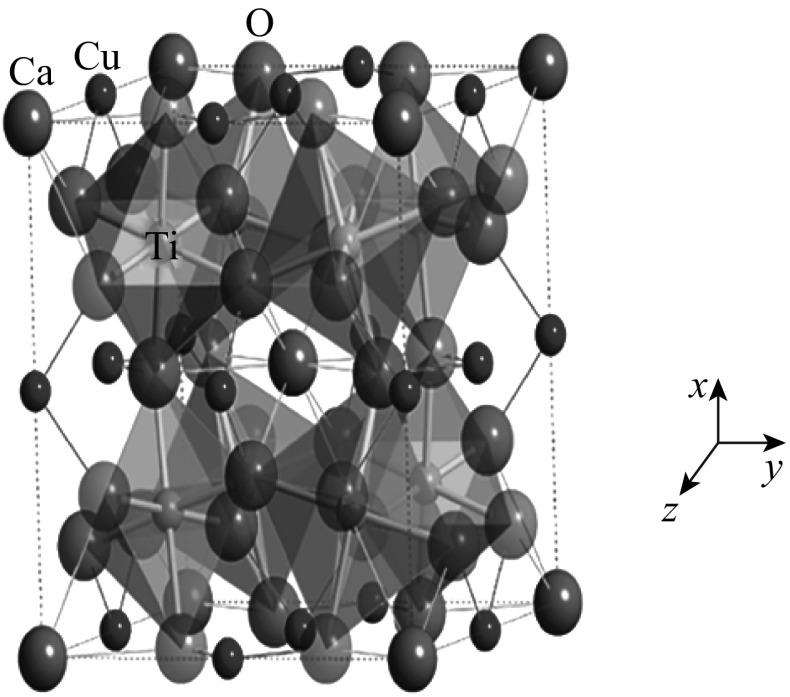
A crystalline structure of CaCu_3_Ti_4_O_12_. *Large white blue atoms* are Ca, *medium-sized dark blue atoms* are Cu, *red atoms* are O, and atoms in the *octahedra centers* are Ti [43]. (Color figure online)

**Fig. 9:**
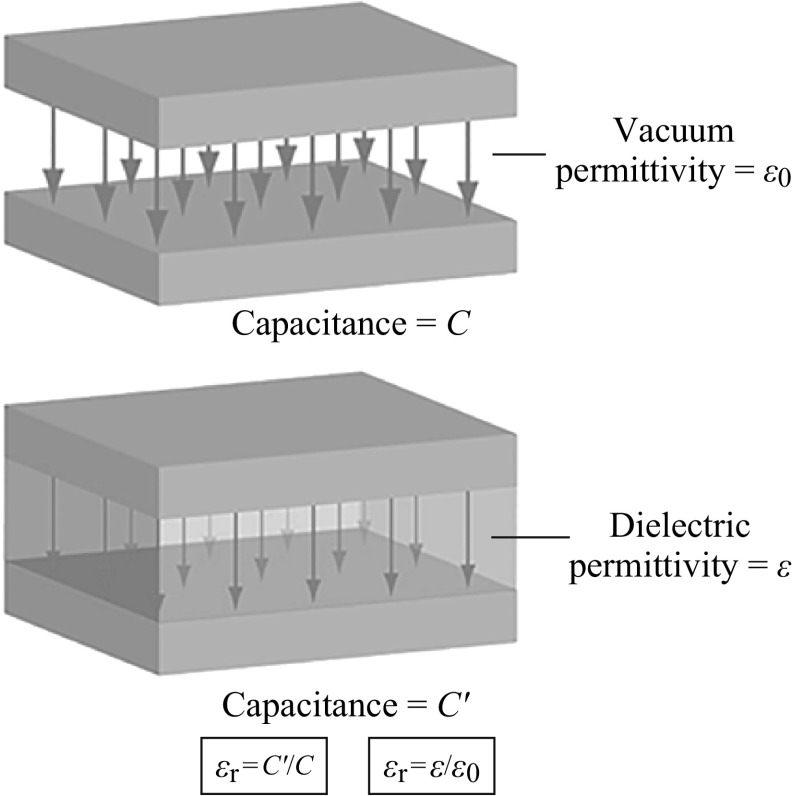
Dielectric permittivity concept

The crystal structure of CCTO [41] shown in Fig. 8 can be obtained from the ideal cubic perovskite structure by superimposing a body-centered ordering of Ca^2+^ and Cu^2+^ ions share in A-site [42]. The size difference between Ca^2+^ and Cu^2+^ causes the TiO_6_ octahedra to undergo remarkable tilting, leading to a body-centered cubic supercell of space group Im^3^. Consequently, the Ti^4+^ ions engrossed centrosymmetric position in the octahedral sites. The tilted angle is so appropriate that the Cu^2+^ ions occupy mostly a square-planer environment [43]. Tilting also significantly changes the coordination environments of the A-site cations which lead to a 4-coordinate square-planar environment for Cu and a 12-coordinate icosahedral environment for Ca. It is the mismatch in size and the bonding preferences of these two ions and the titanium that drive the large octahedral tilting distortion. The Ti^4+^ cations could be displaced off center along their one–threefold axis. However, this cannot be a pure ferroelectric transition, because the displacements occur along four different directions. Thus, CCTO has a perovskite-type structure where *ɛ*_r_ is increased by tension on the Ti-O bonds. Figure 9 illustrates the unit cell of body-centered cubic CCTO with an Im^3^ space group, which consists of two formula units. The Ti atoms sit at the center of the canted TiO_6_ octahedra (the tilt angle is nominally 141°), with bridging Cu atoms bonded to the oxygen atoms, and with large Ca atoms at the corners and center of the unit cell.

Two main features are needed for any dielectric material in practical applications: high *ɛ*_r_ and low tan *δ*.

### Dielectric Permittivity

The *ɛ*_r_ is the ratio of the capacitance of a capacitor filled with the given material to the capacitance of an identical capacitor in a vacuum without dielectric material. The insertion of a dielectric between the plates of a parallel-plate capacitor always increases its capacitance, or ability to store opposite charges on each plate, compared with the ability when the plates are separated by a vacuum. If *C′* is the value of the capacitance of a capacitor filled with a given dielectric and *C* is the capacitance of an identical capacitor in a vacuum which is showed in Fig. 9. The *ɛ*_r_ is dimensionless, and simply expressed as *ɛ*_r_ = *C′*/*C*. It denotes a large-scale property of dielectrics without specifying the electric behavior on the atomic scale.

The *ɛ*_r_ of CCTO has been reported to range from 100 to 300,000 in pellets, films, single crystals, and polycrystalline microstructures [44–46]. The highest value of *ε* remains essentially unchanged ranging from 100 to 400 K. A completely satisfactory explanation is not yet available for this unique and remarkable behavior. However, it is established that the behavior is not related to the ferroelectric transition [47], and the consensus is that the behavior is not intrinsic [48]. According to a well-established barrier layer mechanism for high *ɛ*_r_, the high *ε* values of CCTO have been ascribed as being due to the conducting grains with insulating grain boundaries and its possible source. [49]. However, the exceptionally high *ε* values in CCTO crystals dictates that the insulating barriers must be inside the crystals rather than between them. Very high concentrations of twin boundaries seem always to be present in CCTO crystals. It has been suggested that these twin boundaries somehow act as the insulating barriers. An understanding of the dielectric properties of CCTO requires an explanation for the manner in which this titanate develops its conducting regions, and a description of the insulating boundaries within the conducting regions. Although the high *ɛ*_r_ of CCTO can be rationalized based on its atomic structure, there is a good reason to suspect that the *ɛ*_r_ of this phase is enhanced by its microstructure. The dielectric behavior is scientifically interesting and technologically intriguing. Therefore, the origin of large dielectric permittivity of CCTO has attracted much attentions. So far, several models though controversial have been proposed to explain the dielectric behavior. The mechanism for the giant dielectric constant of CCTO is still questionable, and investigators are studying it in an effort to understand whether the giant dielectric constant is intrinsic to a perfect crystal or extrinsic [50].

### Dielectric Loss

Tangent δ (tan *δ*) or energy loss in a dielectric is due to an alternating electric field, which is a material property rather than geometry property of a capacitor. Usually the tan *δ*, expressed as the dissipation factor (*D*_f_) or loss tangent (tan *δ*) can be mathematically defined as per the Eq. 3:

3 $${ \tan }\delta = \frac{\varepsilon^{\prime \prime} }{\varepsilon^{\prime} }$$

where the angle *δ* is supplementary angle of the phase difference between the applied electric field and the induced current. $\varepsilon^{\prime \prime}$ and $\varepsilon^{\prime}$ are the real and the imaginary parts of the *ɛ*_r_. The value of tan *δ* has been found to be about 0.115 at 1 MHz at room temperature [51]. Therefore, the CCTO, which presents a large *ɛ*_r_, fails to meet the second requirement. To reduce the dissipation factor (*D*_f_) by keeping the *ɛ*_r_ constant, different methods were employed. These include the techniques such as applying different sintering methods or doping additional oxides. In general, dielectric loss of a dielectric material results from distortional, dipolar, interfacial, and conduction loss. The distortional loss is related to electronic and ionic polarization mechanisms. The interfacial loss originates from the excessive polarized interface induced by the fillers, and specifically the movement or rotation of the atoms or molecules in an alternating electric field. The conduction loss is attributed to the *dc* electric conductivity of the materials which represents the flow of actual charge through the dielectric materials.

### Factors Affecting the Property and Morphology of CCTO

Like the influences of doping on CCTO, it seems that different processing conditions can significantly affect the dielectric properties as well. For CCTO, Bender and Pan [52] examined the effects of the processing conditions in detail by using various conditions including the powder mixing, firing temperature (both calcination and sintering), and annealing. They revealed that the *ɛ*_r_ increased when the CCTO powder was mixed via a milling method accompanied with a higher sintering temperature and a longer sintering time. They also suggested that improvement of *ɛ*_r_ can be attributed to higher concentration of defects in the grain core.

#### Effect of Sintering Temperature and Time

Sintering of CCTO electroceramic is the method that involves heating the CCTO powder green compact part to a high temperature below the melting point when the material of the separate particles diffuse to the neighboring powder particles. The driving force of sintering process is reduction of surface energy of the particles caused by decrease in their vapor–solid interfaces. The reduction in energy is accomplished by an atomic diffusion process. The process leads to densification of the body by transporting matter from inside the grains into the microstructures of pores of the matter between different parts of the pore surfaces leading to a decrease in the pore volume. Sintering process may be conducted in different atmospheres: inert, and air atmosphere.

**Fig. 10:**
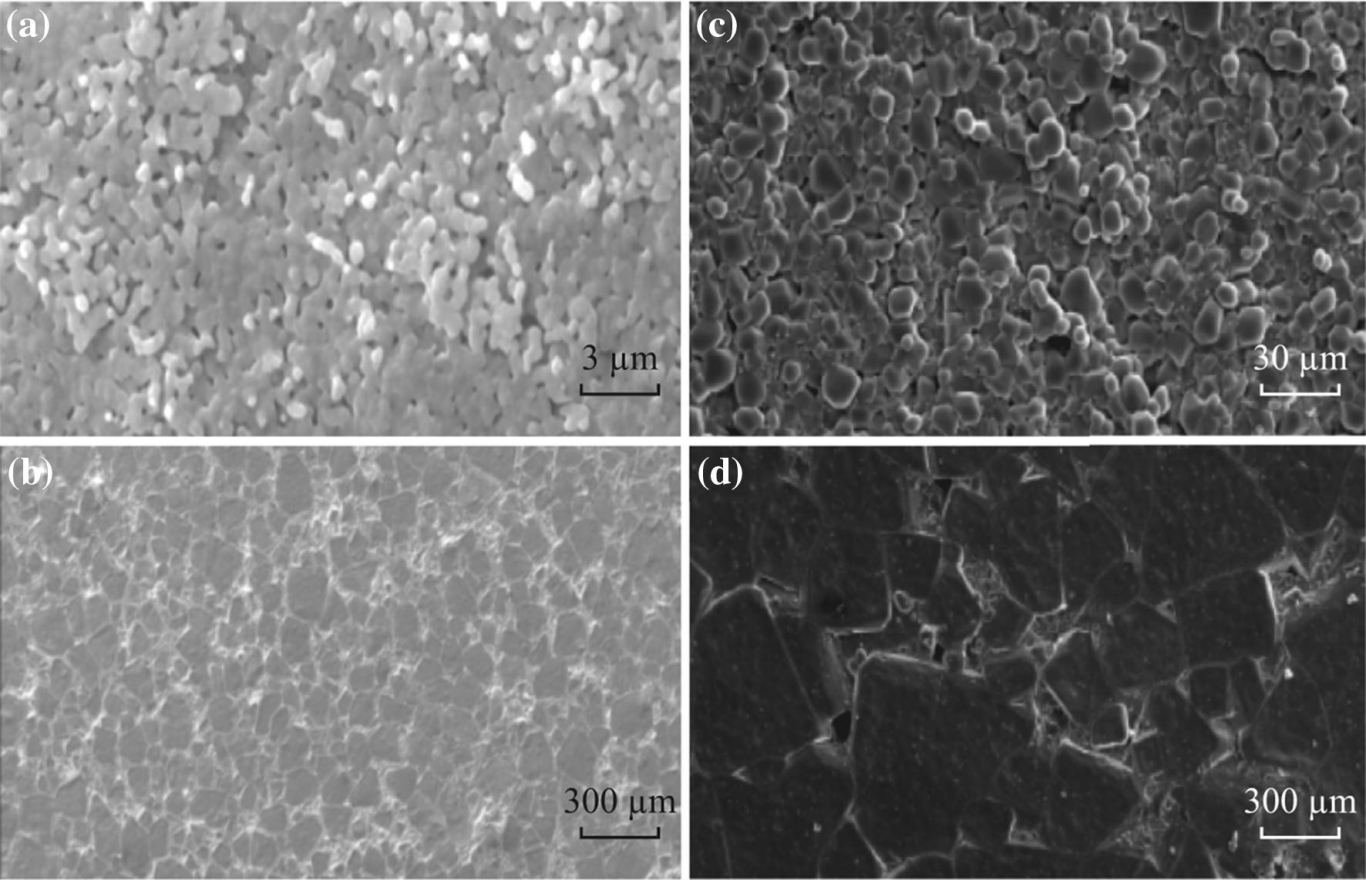
SEM images of ceramic microstructure for CCTO ceramics sintered for 3 h for **a** 900 °C, and **b** 1050 °C. CCTO ceramics sintered at1100 °C for 3 h (**c**) and 24 h (**d**)

Typically, Sinclair et al. [53] reported that the impedance measured at 1 kHz showed great enhancement of *ɛ*_r_ from ~7700 of 900 °C to ~60,000 of 1050 °C at 3 h, as illustrated in Table 2. Consequently, the temperature shows a compelling effect on the CCTO grain size, as shown in Fig. 10a, b. It is also obvious that with the increasing grain size, there is corresponding decrease in the total volume faction of boundary layer of the CCTO. Further, the overall permittivity decreases because of the total number of capacitors that are constructed by the microlayers’ increases, whereas the total conductivity decreases as some portion of the insulating boundary layer vanishes. Furthermore, time has a characteristic effect on the grain size of CCTO with consequent effect on the *ɛ*_r_. The grain size reported for the CCTO electroceramic increases from less than 30 µm to around 300 µm as shown in Fig. 10c, d. The value of *ɛ*_r_ increases from ~9000 to ~280,000 at 3 h and 24 h, respectively, for CCTO ceramics sintered at 1100 °C, as revealed in Table 3. The *ɛ*_r_ obviously increases with the sintering temperature and time as shown in Tables 2 and 3. It is also accordingly closely related to the polycrystalline microstructure, particularly to the grain sizes.

**Table 2:** Effects of sintering temperatures on dielectric permittivity of CCTO

Time (h) (constant)	T (°C)	*ɛ*_r_ (at 1 kHz)	References
3	900	7700	[53]
	1050	60,000	[53]
5	1040	5000	[55]
	1060	84,600	[55]
10	1000	1200	[27]
	1120	60,000	[27]
12	1000	1200	[56]
	1025	5500	[56]
	1100	100,000	[56]

**Table 3:** Effects of sintering times on dielectric permittivity of CCTO

T (°C) (constant)	Time (h)	*ɛ*_r_ (at 1 kHz)	References
1100	3	9000	[53]
	24	280,000	[53]
1100	2	16,000	[57]
	8	30,000	[57]
	33	59,000	[57]
1000	4	1000	[58]
	6	1900	[58]
	8	3900	[58]
1050	3	20,000	[59]
	20	40,000	[59]
1120	3	514	[60]
	6	12,400	[60]

#### Effect of Doping

A doping method opens an effective way to alter the electric performance, both high *ɛ* and low tan *δ* are essential for different applications. The dielectric material that has a low dielectric loss and low *ɛ*_r_ is useful in insulators. However, high *ɛ*_r_ and low tan *δ* are desirable for a capacitor component. Co^2+^/Co^3+^ doping was studied by Chiodelli et al. [61]. The adopted doping dramatically increased the *ɛ*_r_ value of CCTO as shown in Table 4. Especially in the case of 5 % Co doping, the *ɛ*_r_ was raised to 147,000, which is 15 times larger than that of the undoped CCTO.

**Table 4:** Effects of doping on CCTO

Material	Concentration	*ɛ*_r_ (10^3^ Hz)	tan *δ* (10^2^–10^4^ Hz)	Grain size	References
Al^3+^	0.3	16,000	~0.1	~5 µm	[62]
	0.06	70,000	<0.06	47 µm	[63]
Nb^5+^	0.1	400,000	<0.2	7 µm	[64]
	0.2	420,000	2 ≤ *x* ≤ 0.4	7 µm	[64]
Sb^5+^	0.05	24,000	1.5 ≤ *x* ≤ 0.2	20 µm	[65]
	0.025	20,000	1 ≤ *x* ≤ 0.2	30 µm	[65]
Zn^2+^	0.2	12,500 ≤ *x* ≤ 2500	1.27 ≤ *x* ≤ 0.1	1 µm	[66]
	0.05	15,000	0.029	~1 µm	[67]
Pr^3+^/Pr^4+^	0.2	4500	0.4 ≤ *x* ≤ 0.1	5 µm	[68]
	0.05	3500	0.15 ≤ *x* ≤ 0.1	4 µm	[68]
Sr^2+^	0.2	14,348	0.7 ≤ *x* ≤ 0.08	–	[69]
	0.1	14,369	0.08 ≤ *x* ≤ 0.04	–	[69]
Fe^3+^	0.2	100	9.6 ≤ *x* ≤ 1	~120 µm	[70]
	0.03	433	5 ≤ *x* ≤ 1	–	[71]
Ni^2+^	0.02	2500	0.15	4 µm	[72]
	0.2	10,000	~0.6	–	[73]
Y^3+^	0.02	2700	0.06	200 nm	[74]
	0.1	75,000	<0.2	93 µm	[75]
B^3+^	0.03	50,000	<0.1	12 µm	[76]
	0.01	~50,000	0.16 ≤ *x* ≤ 0.09	10 µm	[76]
Te^2+^	0.02	20,000	0.1 ≤ *x* ≤ 0.05	3.23 µm	[77]
	0.01	25,500	0.12 ≤ *x* ≤ 0.05	2.51 µm	[77]
Co^2+^/Co^3+^	0.4	9500	1.3 ≤ *x* ≤ 0.5	5 µm	[78]
	0.2	70,000	≤ 0.6	~5 µm	[78]
Zr^4+^	0.1	33,000	1.6 ≤ *x* ≤ 0.2	~10 µm	[79]
	0.05	15,000	2.4 ≤ *x* ≤ 0.2	5 µm	[80]
Ga^3+^	0.05	38,011	~0.1	136 µm	[81]
	0.1	66,736	0.15 ≤ *x* ≤ 0.1	199 µm	[81]
La^3+^	0.05	8000	0.6 ≤ *x* ≤ 0.3	–	[82]
	0.2	11,000	~0.2	2 µm	[83]
Mg^2+^	0.05	10,000	~0.2	10.5 µm	[84]
	0.1	5000	~0.2	~6 µm	[84]
Sm^3+^	0.005	10,000	0.5 ≤ *x* ≤ 0.03	–	[85]
	0.01	1200	0.1 ≤ *x* ≤ 0.05	–	[85]
Mn^3+^/Mn^4+^	0.06	45	–	–	[86]
	0.01	22,500	~0.5	–	[87]
Sc^3+^	0.08	80,000	1.1 ≤ *x* ≤ 0.1	30 µm	[88]
	0.2	30,000	3 ≤ *x* ≤ 0.3	10 µm	[88]
Ba^2+^	0.05	8000	~0.2	5.79 µm	[89]
	0.2	1500	0.27 ≤ *x* ≤ 0.23	~1 µm	[90]

The reasons for the observed wide variation in dielectric properties for CCTO are unknown: it is because the nature of its giant permittivity is still open to scientific debate.

Hong et al. [64] studied the effects of isovalent dopant Nb on the dielectric properties of CCTO. The CCTO was doped with different concentrations of Nb^5+^ (*x* = 0, 0.02, 0.1, 0.2, 0.4). They found that the dopant concentration has profound effect on the *ɛ*_r_ of CCTO. The resistivity of boundary layers and *ɛ*_r_ of the CCTO increase with the increasing dopant concentrations.

XRD was utilized in their work to observe changes in lattice parameters and phase evolution. The XRD data showed a modest linear increase in lattice parameters with the addition of dopant up to *x* = 0.4. Therefore, they concluded that the added Nb^5+^ was most likely present in the microstructure either in the grain boundaries or as a secondary phase. The XRD data also showed that there was no secondary phase as the grain size reduced to ~7 µm with *x* = 0.1 Nb^5+^ substitution.

Vangchangyia et al. [69] synthesized Ca_1−*x*_Sr_*x*_Cu_3_Ti_4_O_12_ (*x* = 0.05, 0.1, 0.15, 0.2) ceramics using a simple thermal decomposition method and studied their dielectric properties. It was found that by increasing the Sr^2+^ concentrations from 5 to 15 at%, there was slight decrease in tan *δ* (<0.04). However, *ε*_r_ was reduced with its value being still higher than10^4^.

Jin et al. [83] examined the effect of La^3+^ on the compositions of Ca_1−*x*_La_2*x*/3_Cu_3_Ti_4_O_12_. It was found that these ceramics show enhancement in *ε*_r_ value (1700–1,1000 at 10^3^ Hz) and sufficiently low tan *δ* (<0.2). Over the frequency range from 10^2^ to 10^5^ Hz, tan *δ* values at *x* = 0.05–0.20 were small, and almost did not vary with frequency. La^3+^ doping plays important roles in the observed excellent dielectric properties in Ca_1−*x*_La_2*x*/3_Cu_3_Ti_4_O_12_ ceramics.

## Deposition Techniques of CCTO Thin Films

Many researchers focused on the preparation of CCTO thin films [91–93] due to their unusual dielectric properties and potential applications for microelectronic devices. The relationship between the dielectric properties and microstructures of the deposited thin films was studied in terms of the different substrates via pulsed laser deposition (PLD) [92, 94–96] or sputtering [97] to metal organic chemical vapor deposition (MOCVD) [98, 99] or sol–gel [100–102].

### Pulsed Laser Deposition (PLD)

The PLD technique is very effective and well suited for developing epitaxial films, and allows for fabrication of multilayers, heterostructures, and super lattices. The technique was first used by Smith and Turner in 1965 for the preparation of semiconductors and dielectric thin films [103]. The first research on CCTO thin films was published in the IEEE proceedings by Cho et al. [92]. Adoption of PLD method caused ceramic thin films to grow on substrates like LaAlO_3_ (LAO) (100) and SrRuO_3_. A study about the PLD parameters to obtain optimized properties in epitaxial CCTO films and comparison between these films and polycrystalline thin films was also done. They concluded that the dielectric responses and mechanisms were similar in both cases; i.e., for both cases, the dielectric behavior can be modeled by series combination of two parallel *R*-*C* circuits. The gigantic relaxation process in oriented epitaxial thin-film CCTO was proposed to be caused by the mutual interaction of the domain volume resistance and the domain boundary capacitance. Domain boundaries are likely to be at twin boundaries within the oriented epitaxial thin CCTO film.

Fang et al. [94] reported about obtaining results comparable to epitaxial thin films with thin films produced by PLD on Pt/Ti/SiO_2_/Si substrate. The influence of PLD parameters was studied, and the best films were obtained under oxygen pressure of 26.6 Pa and 720 °C. The values obtained were 2000 for *ɛ*_r_ at 10 kHz and below 0.5 for the tan *δ*. Fang et al. [104] also studied the influence of multilayers CaTiO_3_/CCTO and CaTiO_3_/CCTO/CaTiO_3_. Samples were produced by PLD with thicknesses of 8–24 nm for CaTiO_3_ (CTO) and 500 nm for CCTO. Simple CCTO was made for comparison purpose, and the results showed that loss is largely reduced by the introduction of CTO buffer layers, while *ɛ*_r_ value also increased. At 10 kHz, the *ɛ*_r_ of CCTO was about 1005. The *ɛ*_r_ value for CTO/CCTO/CTO was about 1507 (w/CTO 16 nm). Besides, the tan *δ* of CCTO was 0.17, while the tan *δ* for the double-buffered was 0.106. The authors also presented a study about the introduction of a SiO_2_ layer with different thicknesses between two layers of CCTO [105]. Films were obtained also by PLD technique. They reported the lower value of tan *δ* and the leakage current density of the multilayer thin films with decreased value of *ɛ*_r_. Two reasons were pointed out to explain this behavior: one was the improvement in the crystallinity, and the other was the reduction of the free carriers in the multilayered films. Multilayer films with 20-nm SiO_2_ layer showed a tan *δ* of 0.065 at 100 kHz and a value of *ɛ*_r_ of approximately 150. The main drawbacks of PLD are an inhomogeneous energy distribution in the laser beam profile, which consequently gives rise to an inhomogeneous energy profile and angular energy distribution in the laser plume. Due to the involvement of the high laser energies, macroscopic and microscopic particles from the target can be ejected. This effect is detrimental to the desired properties of films and multilayers [94].

Deng et al. [96] investigated experimentally the relationship between the electric properties and the oxidation states of Cu and Ti in CCTO thin films deposited by pulsed laser deposition (PLD) on top of 150-nm-thick SrRuO_3_ conductive layer on top of a LaAlO_3_ single crystal. Their results demonstrated that the as-deposited CCTO film was made of insulating grain boundaries with semiconducting grains, indicating that the high dielectric-constant can be attributed to barrier layer capacitor (BLC) effects. The capacitance of the grains and grain boundaries can be tuned by changing the annealing atmosphere and temperature. Under an oxygen-absent annealing atmosphere, the electric resistances of the grain boundaries changed greatly, but the resistance of the grains showed almost no change, while under an oxygen-annealing atmosphere, the reverse process occurred. On the basis of this result, it is demonstrated that the origin of the dielectric response of the grains in CCTO films is attributed to their oxygen-loss, while the grain boundaries are close to oxygen-stoichiometry. The high apparent *ɛ*_r_ value in the CCTO thin film can be suppressed by annealing the sample in air.

### RF Sputtering

Prakash et al. [97] reported high-quality films with preferential (220) orientation obtained at a substrate temperature of 650 °C under a total pressure of 4.86 Pa with 1 % O_2_. The *ɛ*_r_ was reported to be ~5000 at 1 kHz and 400 K. Also, the frequency of the dielectric relaxation in thin films was found to be much lower than that observed in bulk ceramics, and the dielectric relaxation was much higher.

### Metal Organic Vapor Deposition (MOCVD)

Nigro et al. [98] analyzed microstructural thin films of CCTO prepared by MOCVD on LaAlO_3_ substrates. Unlike other referenced works in this review, they did not report the dielectric response of the CCTO. However, they asserted that the deposited MOCVD films consisted of CuO grains embedded in a quite amorphous matrix of Ca–Ti oxides. After the in-situ annealing step at 900 °C, CCTO phase was formed, and the XRD patterns showed the formation of (100)-oriented CCTO films. In the case of LaAlO_3_ (100) CCTO films, it was found that 1100 °C treatment for 24 h resulted in a very rough and porous surface. In contrast, the same annealing treatment carried out on amorphous CCTO samples revealed the formation of large, rounded grains (600–1000 nm) that were homogenously distributed on the surface. However, after rapid thermal annealing processes at 1100 °C, substantial and flat grains (about 5 μm) were observed, which were similar to those of CCTO ceramics with very high *ɛ*_r_ values. Evidences of the formation of CCTO and CaTiO_3_ phases were also reported for the films. The group reported in another paper [100] the influences of low *ɛ*_r_ (low-*k*) layers of SiO_2_ and Si_3_N_4_ on CCTO morphological properties without conducting dielectric characterization. SiO_2_ showed better crystallinity than Si_3_N_4_ due to oxide nature of the buffer layer.

Fiorenza et al. [106] also presented an overview from the process of growing thin films of CCTO to the assessment of the permittivity. Hot-wall MOCVD technique was used to obtain thin films on LaAlO_3_ (001) substrates. The microscopic property was investigated using atomic force microscope (AFM) equipped with the scanning capacitance microscopy module used in scanning impedance configuration. The investigation demonstrated the presence of a surface-depleted layer at the electrode/CCTO film interface, and simultaneously a huge *ɛ*_r_ value of 8000 was measured as an extrinsic local behavior. The absence of the barrier at the macroscopic scale for the films thermal treated at 900 °C was explained by the presence of conducting leaking regions on the films. However, the presence of Schottky barrier was observed for the larger grains of the sample heat treated at 1000 °C with the rising of a local colossal permittivity.

### Sol–Gel

A sol–gel is another method which yields thin film layers. Compared to other methods, sol–gel methods offer several advantages such as (i) good physical and mechanical strength, (ii) low swelling capacity of the material in aqueous or organic solvents, (iii) high chemical inertness, (iv) lower temperatures for processing, and (v) high thermal and photochemical stability. Furthermore, sol–gel-based films require low immobilization temperatures which result in the preservation of the properties of the CCTO thin film. Although the sol–gel process is known for over 150 years, interest in the process began only in the 1980s after some studies showed the possibility of incorporating organic molecules and active proteins in porous ceramic matrices.

Jiménez et al. [107] achieved appreciable electric properties in CCTO films obtained by sol–gel method using a non-methoxyethanol route. Solution was produced using a titanium diol-based precursor, obtained by refluxing of Ti (IV) with 1,3-propanediol in the ratio of 1:1 with two solutions of copper acetate and calcium acetate in 2-ethyl-hexanoic acid in the ratio of 1:10. The group reported tan *δ* with maximum values in the range of 0.2–0.5, and *ɛ*_r_ values in the range of 200–400 at room temperature, which depended on the frequency for spin-coated films heat treated at 650 °C. Moreover, Shen et al. [108] studied the switching resistance characteristics dependence on the annealing parameters of thin CCTO films obtained by a sol–gel on silicon-based substrates. Solution without methoxyethanol was composed of calcium and cupric acetates, acetic acid, titanium isopropoxide, ethylenglycol, and formamide. The CCTO films showed resistance switching phenomena when annealed at 700 °C and above. With the increasing annealing temperature, the crystallinity of the films improved. No report on the dielectric properties of these films was published.

Consequently, reliable results were reported for CCTO films prepared by physical deposition methods as sputtering and PLD. For these techniques, *ɛ*_r_ was measured to be 6000 for epitaxial films, 2000 for polycrystalline ones with PLD, and 5000 for polycrystalline sputtered thin films. The tan *δ* values reported for these techniques were in the range of 0.5–0.2 for PLD, whereas no values were found for sputtered films. Compared to chemical solution deposition methods, these results are very good, but those techniques used are very expensive and complex with time-consuming procedures. In addition, it is difficult to control the stoichiometry of the films. Within the chemical solution deposition methods, the sol–gel [109, 110] and MOCVD have been the most preferred methods used to prepare CCTO thin films.

## Application of CCTO Thin Films as Sensors

Semiconducting metal-oxide (SMO) sensors are among the most widely studied groups of chemiresistive gas sensors. These sensors are designed to react with one class of gases whereby the SMO undergoes reduction and oxidation. This process causes the SMO sensors to exchange electrons with the target gas at a certain characteristic rate, thereby affecting the sensor’s resistance and yielding a certain signal. The reaction of SMO materials with gases and the result of the conductometric changes were introduced in the early 1950s by Brattein et al. [111]. The direct applications of the SMO sensors as catalysts and electric conductive detectors toward various gases were then introduced by Seiyama et al. [112].

During the past few decades, SMO gas sensors have become a prime technology in many industrial gas-sensing systems. Gas sensor technologies are still evolving and yet to reach their full potential in terms of capabilities and usage. Gas sensors based on metal-oxide thin films, such as MgO, Cr_2_O_3_, NiO, SrO, In_2_O_3_, GeO_2_, Nb_2_O_5_, Ta_2_O_5_, and La_2_O_3_, [113], are commonly used in the monitoring of humidity. Gases are based on electrochemical behavior, catalytic combustion, or resistance modulation of SMO [114–120] and can provide the necessary sensitivity and stability required by such system. However, it has difficulties in the measurement of electric conductivity because those materials have large band gaps thereby causing easy formation of electrons and holes. For this reason, CCTO pioneered by Subramanian et al. [1] is being used to improve the sensor properties.

**Fig. 11:**
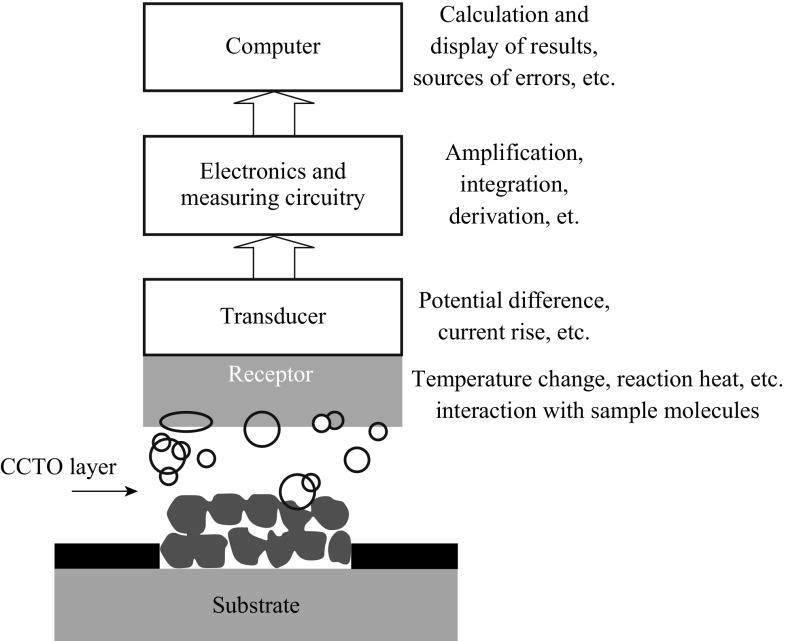
Schematic representation regarding how an appropriate CCTO-based electrochemical sensor works

A CCTO-based sensor—that only few researchers’ have worked on—can be defined as an analytic device, which consists of an immobilized layer of CCTO on a solid support. If the structures and properties of the CCTO are preserved after immobilization, it will recognize the analyte. The chemical reaction between the immobilized CCTO and analyte will then be transformed into an electric signal, which will be amplified and converted by signal-processing equipment, into a display (Fig. 11). Chemical sensors should transform chemical quantities into electric signals. Chemical sensor should respond exclusively to one analytic, or at least be selective to a group of analyses.

### Sensing Mechanism

Considering the influential factors on gas-sensing properties of metal oxides, it is necessary to know the sensing mechanism of a metal-oxide gas sensor. The exact fundamental mechanisms that cause a gas response are still adaptable, but the main responsible reason for conductivity is the trapping of electrons at adsorbed molecules by charged molecules.

**Fig. 12:**
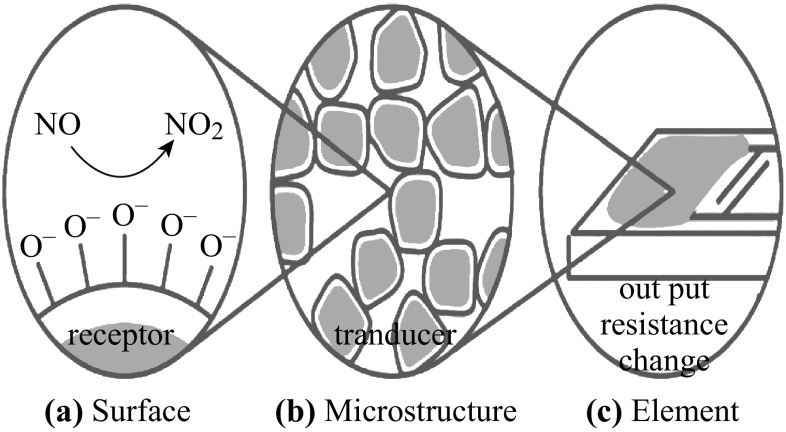
**a** Chemisorption and reaction between reducing gases and oxygen adions (O^−^) at the surface give rise to the receptor function. **b** Electronic charge transport through the grains and across grain boundaries gives rise to the transducer function. The latter depends on the microstructure of the sensing layer, e.g., on the grain size and pore size. **c** The sensor element comprises the sensing layer, electrodes for electric measurements, substrate, and integrated microheater [125]

Figure 12 illustrates the surface chemical reaction mechanism of CCTO gas sensor upon exposure to reference gas with gas analytes and the microstructure of the sensor on the electric response to the gas [121]. The surface chemical reaction can be referred to as the receptor function of the sensor, because it converts a chemical reaction with the gas analyte into a charge transfer event. This motivates an electric signal, which is amplified by the charge transport mechanism through the sensor or the so-called transducer function of the sensor [122–124].

The important factor that influences the performance of CCTO as a gas sensor is the environmental humidity. However, mechanisms of sensing water vapor and other pollution gases are different. For CCTO, humidity-gas sensors and ionic-type humidity sensors are the most commonly used patterns. The conduction mechanism depends on H^+^ or H_3_O^+^, from dissociation of adsorption water [126]. The reaction between the surface oxygen and the water molecules conduces to a decrease in baseline resistance of the gas sensor, and results in a decrease of the sensitivity [127]. Second, the adsorption of water molecules leads to less chemisorption of oxygen species on the sensing layer due to the decrease of the surface area that is responsible for the sensor response. Thus, the sensitivity decreases, and the response and recovery times increase.

### Sensor Properties

**Fig. 13:**
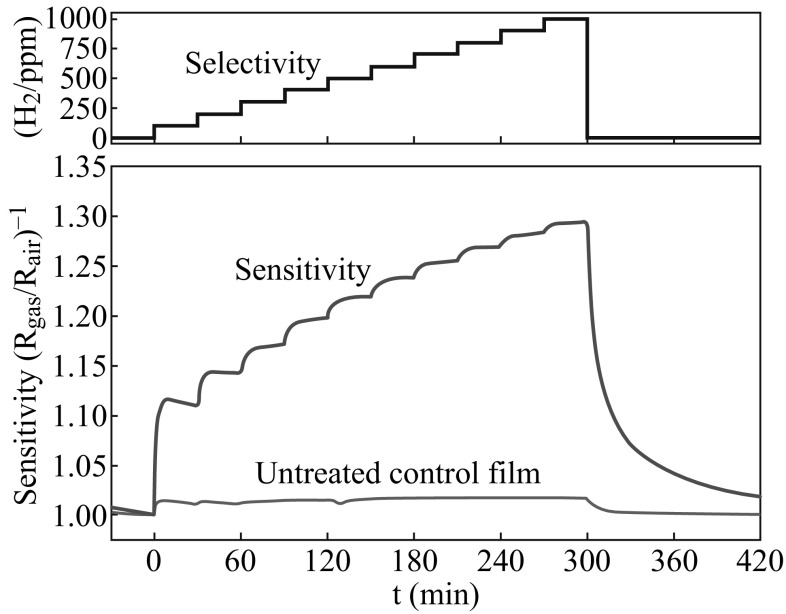
Main characteristics of CCTO-based sensor constructed and deposited on Al_2_O_3_ substrates with interdigitated Pt electrode and Amperometric response showing the sensitivity and selectivity of the sensor to successive injections of hydrogen [16]

Two important factors in sensor properties are sensitivity and selectivity [128–132]. In general, sensitivity depends on the sensor size, activity of the deposited CCTO toward the specific analyte, success in CCTO immobilization by retaining as much activity as possible [16], and also dopant [116]. Figure 13 illustrates examples of these characteristics for a Al_2_O_3_ substrate coated with CCTO. The selectivity deals with the study of interferences that result mostly from the presence of electroactive species in the test medium. A CCTO-based sensor will be selective if the response to the substrate is high and the response to interferences is low. However, the selectivity of sensors for a given gas in a mixture of two or more reactive gases is debatable.

**Fig. 14:**
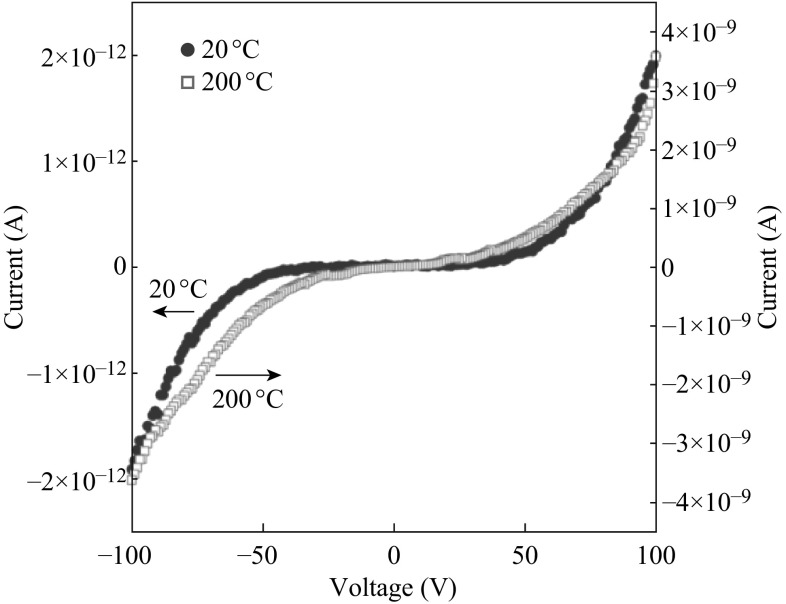
*I*–*V* characteristics for CCTO-based sensor at temperature 20 and 200 °C [16]

On the other hand, the nonlinear response of a sensor should result from the slow rate of oxidation or reduction of the CCTO in the presence of the analyte. Highly nonlinear *I*-*V* characteristics with strong thermal activation were observed, as shown in Fig. 14. Similar nonlinear behavior has been reported recently for polycrystalline CCTO ceramics by Chung et al. [133].

**Fig. 15:**
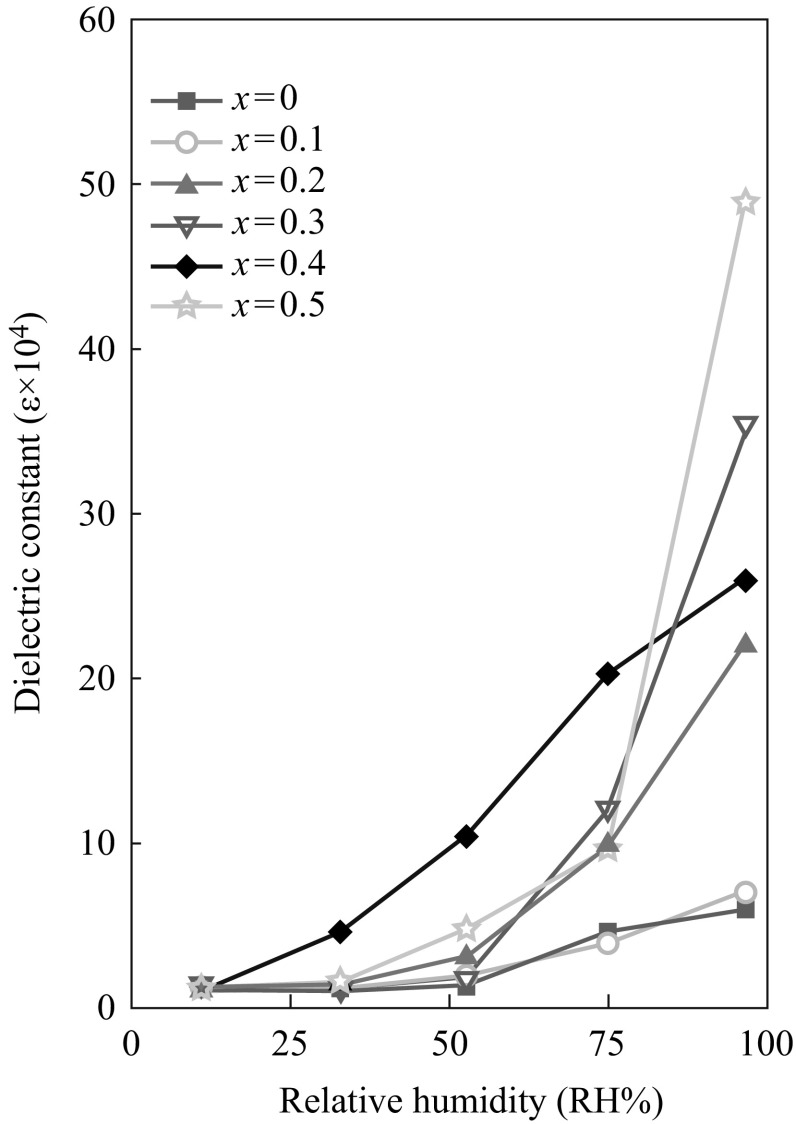
The humidity sensing characteristics of CaCu_3−*x*_MgxTi_4_O_12_ (*x* = 0, 0.1, 0.2, 0.3, 0.4, and 0.5) [127]

Li et al. [127] investigated the effect of addition of Mg to CCTO ceramic-type humidity sensors, which resulted in improved sensitivity and durability as well as in decreased hysteresis. However, sensitivity of doping enhanced CCTO can be seen in Fig. 15.

Gas-sensing properties have also been reported by few researchers for CCTO thin films; the mechanism of a sensor signal is based on conductivity of the CCTO thin film but the type of conductivity in thin films are not well defined in the literature. Kim et al. [16] reported the preparation of macroporous CCTO thin films by PLD onto PMMA (polymethyl methacrylate) microsphere-templated substrates. These films have n-type conductivity and higher H_2_ sensitivity compared to CO and CH_4_. N-type conductivity was also reported by Parra et al. [134], in mesoporous thin films prepared by the sol–gel method. However, Joanni et al. [17] showed that films prepared by rf-sputtering display p-type conductivity. Depending on the synthesized method and experimental conditions, n- or p-type conductivity can be attained. Some of the representative developments in this area are summarized in Table 5.

**Table 5:** Compilation of sensors based on CCTO applied for gas monitoring

Sensor	Method	Material	T (°C)	Thickness	Mechanism	Analytical condition	Response time (min)	Sensitivity	Concentration (measured)	References
H_2_	Pulsed laser deposition (PLD)	CCTO + Al_2_O_3_ substrate +Pt electrode	30–600	80–90 nm	Mechanism not established	Lab (test gas)	300	1.25	5 %	[16]
O_2_	RF sputtering	CCTO + Si/SiO_2_/Ti + Pt substrates	100–200	200 nm	P-type conduction	Lab (test gas)	16	1.5	12 %	[17]
H_2_O	Doping by solid-state reaction	Mg-CCTO (pellet)	25	40 µm	Physisorption mechanism and some may be belonging to change in the capacitance of the barrier layer at grain boundary.	Lab (test gas)	13	31	50 %	[127]
O_2_–N_2_	Sol–Gel	CCTO + Si/SiO_2_ substrate +Pt electrode	220–290	200–400 nm	N-type conduction	Lab (test gas)	–	–	–	[134]

Also researchers have reported that CCTO-modified electrodes can catalyze and recognize specific analyte species. In order to do this, CCTO-modified electrodes have to exhibit certain characteristics of sensors including response time, sensitivity, and concentration, which are shown in Table 5. An overview of the literature in this field revealed that most modified electrodes satisfy the criteria of molecular recognition between the immobilized CCTO and the specific analyte species in terms of sensitivity and linear range, in some cases.

Although all described sensors comply with the concept of molecular recognition between the CCTO and the specific analyte, unfortunately, not all of them can be considered as appropriate sensors because most of them lack high selectivity and low response time. These are the most important characteristics that a good sensor should possess and display. Given the great potential of CCTO in sensing, further research and development of these processes will enable better understanding of the mechanisms of the sensing pathways.

### Sensor Testing Setup

**Fig. 16:**
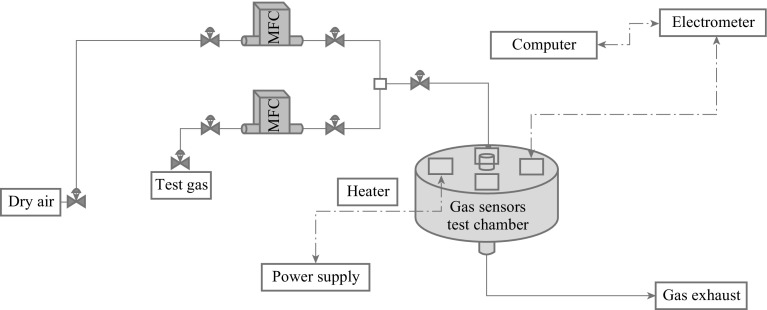
A general schematic for CCTO gas sensor devices

Figure 16 shows the sensor array which mainly consists of a target gas, a multi-component gas mixer, a mass flow controller unit, a testing chamber, a power supplier and heaters, and an electrometer for resistance measurement [121, 135–138]. LabVIEW-based software is mainly used to control all testing parameters and measurements during the experiment. The testing chamber consists of CCTO-based sensor platforms with the ability to control and measure each sensor’s temperature and resistance. The CCTO films are deposited on the sensing element as thin or thick film substrates. Thin film deposits are made via RF sputtering, LPD, and sol–gel techniques. The sensor platform is bonded into a standard header and then placed in a test chamber and temperature adjusted before gas exposure into the chamber.

## Conclusions

The urgent demand for huge *ɛ*_r_ value ceramics has been a key issue leading to the development of many application technologies. In view of this, scientists and technologists have shown the potential of CCTO with high *ɛ*_r_ and low tan *δ* values for such applications. The critical success of new CCTO materials lies in their methods of preparation which controls the morphology, particle size, and its dielectric properties. In order to obtain these features, small particle size, narrow size distribution, uniform morphology, optimum crystallinity degree, high specific surface area, minimum defects, agglomeration, and homogeneous metal doping are required for the practical applications of CCTO powders. The only limitation which restricts the wide range of applications of this compound is the tan *δ*. Methods described in this overview were designed to obtain CCTOs in the form of nano and microcrystalline powders with desired structures and high *ɛ*_r _values. Since the number of surface atoms or ions constitutes a significant fraction of their total number, and the surface energy plays an important role in the crystal structure, the materials in the nanometer size range exhibit some remarkable properties which can be exploited to get CCTO with a high *ɛ*_r_ and low tan *δ*. Besides, the gas-sensing process is strongly related to the surface reactions. Hence, high surface areas can provide large reaction contact area between gas-sensing materials and targeted gases. Porous structure with high surface areas seems to be the standard structure of CCTO gas sensor layers. Fabrication of microstructure-based CCTO for novel generation of future advanced CCTO-based sensors with appropriate properties in terms of sensitivity, detection limit, response time, and stability are required. Adaptability should also be considered because the final goal of all these sensors is the discovery of analytes in real-life analytic, environmental, and biomedical samples. Other causes, such as temperature and humidity, also play important roles in the testing of sensitivity. Humidity will decrease the sensitivity and may be harmful to repeatability. The most important characteristics of these sensors are very high sensitivity, simple signal (resistance), small size, simple constitution, possibility of large-scale manufacturing in very high volumes (these sensors can be manufactured using the microelectronic and MEMS technologies), and easy integration into electronic boards. All these advantages make it a promising technology for the future. These types of sensors can be envisaged to detect polluting gases such as CO, SO_2_, NO_2_, O_3_, fuel gases (hydrocarbon and hydrogen), toxic gases (H_2_S, NH_3_), and volatile organic compounds.

## References

[1] Subramanian MA, Li D, Duan N, Reisner BA, Sleight A (2000). High dielectric constant in ACaCu_3_Ti_4_O_12_ and A CaCu_3_Ti_4_O_12_ phases. J. Solid State Chem..

[2] Lohnert R, Bartsch H, Schmidt R, Capraro B, Topfer J (2014). Microstructure and electric properties of CaCu_3_Ti_4_O_12_ multilayer capacitors. J. Am. Ceram. Soc..

[3] Kretly LC, Almeida AFL, de Oliveira RS, Sasaki JM, Sombra ASB (2003). Electrical and optical properties of CaCu_3_Ti_4_O_12_ (CCTO) substrates for microwave devices and antennas. Microw. Opt. Technol. Lett..

[4] Ponce MA, Ramirez MA, Schipani F, Joanni E, Tomba JP, Castro MS (2015). Electrical behavior analysis of n-type CaCu_3_Ti_4_O_12_ thick films exposed to different atmospheres. J. Eur. Ceram. Soc..

[5] Sulaiman MA, Hutagalung SD, Ain MF, Ahmad ZA (2010). Dielectric properties of Nb-doped CaCu_3_Ti_4_O_12_ electroceramics measured at high frequencies. J. Alloy. Compd..

[6] Yuan WX, Hark SK, Mei WN (2010). Investigation of triple extrinsic origins of colossal dielectric constant in CaCu_3_Ti_4_O_12_ ceramics. J. Electrochem. Soc..

[7] Hua WM, Fu Z, Li WQ, Chao Y (2012). Synthesis of CaCu_3_Ti_4_O_12_ powders and ceramics by sol–gel method using decanedioic acid and its dielectric properties. J. Cent. South Univ..

[8] Banerjee N, Krupanidhi SB (2010). Low temperature synthesis of nano-crystalline CaCu_3_Ti_4_O_12_ through a fuel mediated auto-combustion pathway. Curr. Nanosci..

[9] Kim KM, Kim SJ, Lee JH, Kim DY (2007). Microstructural evolution and dielectric properties of SiO_2_-doped CaCu_3_Ti_4_O_12_ ceramics. J. Eur. Ceram. Soc..

[10] Barbier B, Combettes C, Guillemet-Fritsch S, Chartier T, Rossignol F, Rumeaud A, Lebey T, Dutarde E (2009). CaCu_3_Ti_4_O_12_ ceramics from co-precipitation method: dielectric properties of pellets and thick films. J. Eur. Ceram. Soc..

[11] Ahmad MM, Al-Libidi E, Al-Jaafari A, Ghazanfar S, Yamada K (2014). Mechanochemical synthesis and giant dielectric properties of CaCu_3_Ti_4_O_12_. Appl. Phys. A.

[12] Marques VPB, Ries A, Simoes AZ, Ramrez MA, Varela JA, Longo E (2007). Evolution of CaCu_3_Ti_4_O_12_ varistor properties during heat treatment in vacuum. Ceram. Int..

[13] Cordeiro MAL, Souza FL, Leite ER, Lanfredi AJC (2008). Anomalous current-voltage behavior of CaCu_3_Ti_4_O_12_ ceramics. Appl. Phys. Lett..

[14] Yuana WX, Harka SK, Meib WN (2009). Effective synthesis to fabricate a giant dielectric-constant material CaCu_3_Ti_4_O_12_ via solid state reactions. J. Ceram. Process. Res..

[15] Bueno PR, Varela JA, Longo E (2008). SnO_2_, ZnO and related polycrystalline compound semiconductors: an overview and review on the voltage-dependent resistance (non-ohmic) feature. J. Eur. Ceram. Soc..

[16] Kim I-D, Rothschild A, Hyodo T, Tuller HL (2006). Microsphere templating as means of enhancing surface activity and gas sensitivity of CaCu_3_Ti_4_O_12_ thin films. Nano Lett..

[17] Joanni E, Savu R, Bueno PR, Longo E, Varela JA (2008). P-Type semiconducting gas sensing behavior of nanoporous rf sputtered CaCu_3_Ti_4_O_12_ thin films. Appl. Phys. Lett..

[18] Heiland G (1954). Zum Einfluss von Wasserstoff auf die elektrische leitfähigkeit von ZnO-kristallen. Zeit. Phys..

[19] Bielanski A, Deren J, Haber J (1957). Electric conductivity and catalytic activity of semiconducting oxide catalysts. Nature.

[20] Wang B, Pu YP, Wua HD, Chena K, Xua N (2013). Influence of sintering atmosphere on dielectric properties and microstructure of CaCu_3_Ti_4_O_12_ ceramics. Ceram. Int..

[21] Liu P, Lai Y, Zeng Y, Wu S, Huang Z, Han J (2015). Influence of sintering conditions on microstructure and electrical properties of CaCu_3_Ti_4_O_12_ (CCTO) ceramics. J. Alloy. Compd..

[22] Shao SF, Zhang JL, Zheng P, Zhong WL, Wang CL (2006). Microstructure and electrical properties of CaCu_3_Ti_4_O_12_ ceramics. J. Appl. Phys..

[23] Liu J, Smith RW, Mei WN (2007). Synthesis of the giant dielectric constant material CaCu_3_Ti_4_O_12_ by wet-chemistry methods. Chem. Mater..

[24] Yang Z, Zhang Y, Xiong R, Shi J (2013). Effect of sintering in oxygen on electrical conduction and dielectric properties in CaCu_3_Ti_4_O_12_. Mater. Res. Bull..

[25] Li Y, Liang P, Chao X, Yang Z (2013). Preparation of CaCu_3_Ti_4_O_12_ ceramics with low dielectric loss and giant dielectric constant by the sol–gel technique. Ceram. Int..

[26] Wang MH, Zhang B, Zhou F (2014). Preparation and characterization of CaCu_3_Ti_4_O_12_ powders by non-hydrolytic sol–gel method. J. Sol-Gel. Sci. Technol..

[27] Laijun L, Huiqing F, Pinyang F, Xiuli C (2008). Sol–gel derived CaCu_3_Ti_4_O_12_ ceramics: synthesis, characterization and electrical properties. Mater. Res. Bull..

[28] Surowiak Z, Kupriyanov MF, Czekaj D (2001). Properties of nanocrystalline ferroelectric PZT ceramics. J. Eur. Ceram. Soc..

[29] H.Q. Fan, H.E. Kim, Microstructure and electrical properties of sol–gel derived Pb(Mg_1/3_Nb_2/3_)_0.7_Ti_0.3_O_3_ thin films with single perovskite phase. Jpn. J. Appl. Phys. **41**(11B), 6768–6772 (2002). http://iopscience.iop.org/1347-4065/41/11S/6768

[30] Moussa SM, Kennedy BJ (2001). Structural studies of the distorted perovskite Ca_0.25_Cu_0.75_TiO_3_. Mater. Res. Bull..

[31] Ahmadipour M, Venkateswara Rao K, Rajendar V (2012). Formation of nano scale Mg_(x)_Fe_(1-x)_O (x = 0.1, 0.2, 0.4) structure by solution combustion: effect of fuel to oxidizer ratio. J. Nanomater..

[32] Xanthopoulou G (2010). Catalytic properties of the SHS products. Adv. Sci. Technol..

[33] A.G. Merzhanov, V.V. Barzykin, *Some problems of propellant ignition*. Preprint of the Institute of Chemical Physics of USSR Academy of Science, Moscow, 1970

[34] Gillan EG, Kaner RB (1996). Synthesis of refractory ceramic via rapid metathesis reactions between solid-state precursor. Chem. Mater..

[35] Kingsley JJ, Patil KC (1988). A novel combustion process for the synthesis of fine particle α-alumina and related oxide materials. Mater. Lett..

[36] Ahmadipour M, Hatami M, Rao KV (2012). Preparation and characterization of nano-sized (Mg_(x)_Fe_(1–x)_O/SiO_2_) (x = 0.1) core-shell nanoparticles by chemical precipitation method. Adv. Nanoparticles.

[37] S. Patra, Synthesis and characterization of CaCu_3_Ti_4_O_12_ and lanthanum doped CaCu_3_Ti_4_O_12_ by auto-combustion technique, Master of science thesis, Department of Ceramic Engineering National Institute of Technology, Rourkela, India, 2009

[38] Gendaken A (2003). Sonochemistry and its application to nanochemistry. Curr. Sci..

[39] Wongpisutpaisan N, Vittayakorn N, Ruangphanit A, Pecharapa W (2013). CaCu_3_Ti_4_O_12_ ceramic synthesized by sonochemical-assisted process. Integr. Ferroelectr..

[40] Harvey D (2000). Modern analytical chemistry, illustrated.

[41] Whangbo MH, Subramanian MA (2006). Structural model of planar defects in CaCu_3_Ti_4_O_12_ exhibiting a giant dielectric constant. Chem. Mater..

[42] Adams TB, Sinclair DC, West AR (2006). Characterization of grain boundary impedances in fine- and coarse-grained CaCu_3_Ti_4_O_12_ ceramics. Phys. Rev. B.

[43] Calcium copper tianate. http://en.wikipedia.org/wiki/Calcium_copper_tianate. Accessed 18 September 2015

[44] Mohamed JJ, Hutagalung SD, Ain MF, Deraman K, Ahmad ZA (2007). Microstructure and dielectric properties of CaCu_3_Ti_4_O_12_ ceramic. Mater. Lett..

[45] Fiorenza P, Nigro RL, Bongiorno C, Raineri V, Ferarrelli MC, Sinclair DC, West AR (2008). Localized electrical characterization of the giant permittivity effect in CaCu_3_Ti_4_O_12_ ceramics. Appl. Phys. Lett..

[46] Adams TB, Sinclair DC, West AR (2002). Giant barrier layer capacitance effects in CaCu_3_Ti_4_O_12_ ceramics. Adv. Mater..

[47] Homes CC, Vogt T, Shapiro SM, Wakimoto S, Subramanian MA, Ramirez AP (2003). Charge transfer in the high dielectric constant materials CaCu_3_Ti_4_O_12_ and CdCu_3_Ti_4_O_12_. Phys. Rev. B.

[48] Raevski IP, Prosandeev SA, Bogatin AS, Malitskaya MA, Jastrabik L (2003). High dielectric permittivity in AFe_1/2_B_1/2_O_3_ nonferroelectric perovskite ceramics (A = Ba, Sr, Ca; B = Nb, Ta, Sb). J. Appl. Phys..

[49] Ferrarelli MC, Sinclair DC, West AR, Dabkowska HA, Dabkowski A, Luke GM (2009). Comment on the origin(s) of the giant permittivity effect in CaCu_3_Ti_4_O_12_ single crystals and ceramics. J. Mater. Chem..

[50] Yuan WX, Hark SK (2012). Investigation on the origin of the giant dielectric constant in CaCu_3_Ti_4_O_12_ ceramics through analyzing CaCu_3_Ti_4_O_12_–HfO_2_ composites. J. Eur. Ceram. Soc..

[51] Deschanvres A, Ravenau B, Tollemer F (1967). Remplacement de metal bivalent par le cuivre dans les titanates de type perowskite. Bull. Chim. Soc. Fr..

[52] Bender BA, Pan MJ (2005). The effect of processing on the giant dielectric properties of CaCu_3_Ti_4_O_12_. Mater. Sci. Eng. B.

[53] Sinclair DC, Adams TB, Morrison FD, West AR (2002). CaCu_3_Ti_4_O_12_: One-step internal barrier layer capacitor. Appl. Phys. Lett..

[54] Yuana WX, Li ZJ (2012). Microstructures and dielectric properties of CaCu_3_Ti_4_O_12_ ceramics via combustion method. Eur. Phys. J. Appl. Phys..

[55] Wei L, Zhao-Xian X, Hao X (2014). Preparation and electrical properties of CaCu_3_Ti_4_O_12_ thin ceramic sheets via water-based tape casting. J. Inorg. Mater..

[56] Schmidt R, Stennett MC, Hyatt NC, Pokorny J, Prado-Gonjal J, Li M, Sinclair DC (2012). Effects of sintering temperature on the internal barrier layer capacitor (IBLC) structure in CaCu_3_Ti_4_O_12_ (CCTO) ceramics. J. Eur. Ceram. Soc..

[57] Romero JJ, Leret P, Rubio-Marcos F, Quesada A, Fernández JF (2010). Evolution of the intergranular phase during sintering of CaCu_3_Ti_4_O_12_ ceramics. J. Eur. Ceram. Soc..

[58] Rajabatabar A, Li WL, Bishe OS, Wang LD, Li XL, Li N, Fei WD (2011). Effect of synthesis technique on dielectric properties of CaCu_3_Ti_4_O_12_ ceramic. Trans. Nonferrous Met. Soc. China.

[59] Sun DL, Wu AY, Yin ST (2008). Structure, properties, and impedance spectroscopy of CaCu_3_Ti_4_O_12_ ceramics prepared by Sol–Gel process. J. Am. Ceram. Soc..

[60] Ni WQ, Zheng XH, Yu JC (2007). Sintering effects on structure and dielectric properties of dielectrics CaCu_3_Ti_4_O_12_. J. Mater. Sci..

[61] Chiodellia G, Massarotti V, Capsoni D, Bini M, Azzoni CB, Mozzati MC, Lupotto P (2004). Electric and dielectric properties of pure and doped CaCu_3_Ti_4_O_12_ perovskite materials. Solid State Commun..

[62] L. Shengtao, W. Hui, L. Chunjiang, Y. Yang, L. Jianying, Dielectric properites of Al-doped CaCu_3_Ti_4_O_12_ ceramics by coprecipitation method, in *IEEE, conference proceedings of ISEIM*, pp. 23–26, 2011. doi:10.1109/ISEIM.2011.6826267

[63] Choi SW, Hong SH, Kim YM (2007). Effect of Al doping on the electric and dielectric properties of CaCu_3_Ti_4_O_12_. J. Am. Ceram. Soc..

[64] Hong SH, Kim DY (2007). Electric and dielectric properties of Nb-doped CaCu_3_Ti_4_O_12_ ceramics. J. Am. Ceram. Soc..

[65] Liu Y, Chen Q, Zhao X (2014). Dielectric response of Sb-doped CaCu_3_Ti_4_O_12_ ceramics. J. Mater. Sci.: Mater. Electron..

[66] Singh L, Rai US (2012). Dielectric properties of ultrafine Zn-doped CaCu_3_Ti_4_O_12_ ceramic. J. Adv. Dielectr..

[67] Hutagalung SD, Ooi LY, Ahmad ZA (2009). Improvement in dielectric properties of Zn-doped CaCu_3_Ti_4_O_12_ electroceramics prepared by modified mechanical alloying technique. J. Alloy. Compd..

[68] Xu LF, Qi PB, Song XP, Luo XJ, Yang CP (2011). Dielectric relaxation behaviors of pure and Pr_6_O_11_-doped CaCu_3_Ti_4_O_12_ ceramics in high temperature range. J. Alloy. Compd..

[69] Vangchangyia S, Yamwong T, Swatsitang E, Thongbai P, Maensiri S (2013). Selectivity of doping ions to effectively improve dielectric and non-ohmic properties of CaCu_3_Ti_4_O_12_ ceramics. Ceram. Int..

[70] Mua C, Zhang H, He Y, Liu P (2010). Influence of temperature on dielectric properties of Fe doped CaCu_3_Ti_4_O_12_ ceramics. Phys. B.

[71] Yang Z, Zhang Y, You G, Zhang K, Xiong R, Shi J (2012). Dielectric and electrical transport properties of the Fe^3+^-doped CaCu_3_Ti_4_O_12_. J. Mater. Sci. Technol..

[72] Li T, Chen J, Liu D, Zhang Z, Chen Z, Li Z, Cao XZ, Wang B (2014). Effect of NiO-doping on the microstructure and the dielectric properties of CaCu_3_Ti_4_O_12_ ceramics. Ceram. Int..

[73] Dong X, Qi S, Ke Z, Xing XH, Tao YY, Hong YR (2013). NiO-doped CaCu_3_Ti_4_O_12_ thin film by sol–gel method. J. Inorg. Mater..

[74] Saji VS, Choe HC (2009). Effect of yttrium doping on the dielectric properties of CaCu_3_Ti_4_O_12_ thin film produced by chemical solution deposition. Thin Solid Films.

[75] Luo F, He J, Hu J, Lin YH (2010). Electric and dielectric behaviors of Y-doped calcium copper titanate. J. Am. Ceram. Soc..

[76] L. Shengtao, Y. Yang, W. Hui, L. Jianying, Dielectric properties of B-doped CaCu_3_Ti_4_O_12_ ceramics, in *IEEE, conference proceedings of ISEIM*, pp. 482–485, 2011. doi:10.1109/ISEIM.2011.6826318

[77] Makcharoen W, Tunkasiri T (2013). Microstructures and dielectric relaxation behaviors of pure and tellurium doped CaCu_3_Ti_4_O_12_ ceramics prepared via vibro-milling method. Ceram. Int..

[78] Mu C, Song Y, Wang H, Wang X (2015). Room temperature magnetic and dielectric properties of cobalt doped CaCu_3_Ti_4_O_12_ Ceramics. J. Appl. Phys..

[79] Li WL, Zhao Y, Chi QG, Zhang ZG, Fei WD (2012). Enhanced performance of core-shell-like structure Zr-doped CaCu_3_Ti_4_O_12_ ceramics prepared by a flame synthetic approach. RSC Adv..

[80] Chi QG, Gao L, Wanga X, Lin JQ, Sun J, Lei QQ (2013). Effects of Zr doping on the microstructures and dielectric properties of CaCu_3_Ti_4_O_12_ ceramics. J. Alloy. Compd..

[81] Jumpatam J, Putasaeng B, Yamwong T, Thongbai P, Maensiri S (2013). Enhancement of giant dielectric response in Ga-doped CaCu_3_Ti_4_O_12_ ceramics. Ceram. Int..

[82] Thongbai P, Jumpatam J, Putasaeng B, Yamwong T, Amornkitbamrung V, Maensiri S (2014). Effects of La^3+^ doping ions on dielectric properties and formation of Schottky barriers at internal interfaces in a Ca_2_Cu_2_Ti_4_O_12_ composite system. J. Mater. Sci.: Mater. Electron..

[83] Jin S, Xia H, Zhang Y (2009). Effect of La-doping on the properties of CaCu_3_Ti_4_O_12_ dielectric ceramics. Ceram. Int..

[84] Li W, Qiu S, Chen N, Du G (2010). Enhanced dielectric response in Mg-doped CaCu_3_Ti_4_O_12_ ceramics. J. Mater. Sci. Technol..

[85] Li J, Fu B, Lu H, Huang C, Sheng JW (2013). Dielectric properties of Sm-doped CaCu_3_Ti_4_O_12_ ceramics. Ceram. Int..

[86] Li M, Feteira A, Sinclair DC, West AR (2006). Influence of Mn doping on the semiconducting properties of CaCu_3_Ti_4_O_12_ ceramics. Appl. Phys. Lett..

[87] Kim CH, Jang YH, Seo SJ, Song CH, Son JY, Yang YS, Cho JH (2012). Effect of Mn doping on the temperature-dependent anomalous giant dielectric behavior of CaCu_3_Ti_4_O_12_. Phys. Rev. B.

[88] Meeporn K, Yamwong T, Pinitsoontorn S, Amornkitbamrung V, Thongbai P (2014). Grain size independence of giant dielectric permittivity of CaCu_3_Ti_4-x_Sc_x_O_12_ ceramics. Ceram. Int..

[89] Thongbai P, Vangchangyia S, Swatsitang E, Amornkitbamrung V, Yamwong T, Maensiri S (2013). Non-Ohmic and dielectric properties of Ba-doped CaCu_3_Ti_4_O_12_ ceramics. J. Mater. Sci.: Mater. Electron..

[90] Xu Z, Qiang H, Chen Z, Chen Y (2015). Dielectric behavior of Ba-doped CaCu_3_Ti_4_O_12_ ceramics prepared from citrate-nitrate combustion derived powders. J. Mater. Sci.: Mater. Electron..

[91] Si W, Cruz EM, Johnson PD, Barnes PW, Woodward P, Ramirez AP (2002). Epitaxial thin films of the giant-dielectric-constant material grown by pulsed-laser deposition. Appl. Phys. Lett..

[92] K. Cho, N. Wu, A. Ignatiev, Dielectric properties of CaCu_3_Ti_4_O_12_ thin films, in *Isaf 2002: proceedings of the 13th IEEE international symposium on applications of ferroelectrics*, pp. 187–190, 2002. doi:10.1109/ISAF.2002.1195901

[93] J.R. Li, Dielectric characterization of polycrystalline and epitaxial thin film CaCu_3_Ti_4_O_12_ (CCTO), in *Proceedings of the 7th international conference on properties and applications of dielectric materials*, vol. 3, pp. 1096–1099, 2003. doi:10.1109/ICPADM.2003.1218614

[94] Fang L, Shen MR (2003). Deposition and dielectric properties of CaCu_3_Ti_4_O_12_ thin films on Pt/Ti/SiO_2_/Si substrates using pulsed-laser deposition. Thin Solid Films.

[95] Zhao YL, Pan GW, Ren QB, Cao YG, Feng LX, Jiao ZK (2003). High dielectric constant in CaCu_3_Ti_4_O_12_ thin film prepared by pulsed laser deposition. Thin Solid Films.

[96] Deng G, Yamada T, Muralt P (2007). Evidence for the existence of a metal-insulator-semiconductor CaCu_3_Ti_4_O_12_ junction at the electrode interfaces of thin film capacitors. Appl. Phys. Lett..

[97] Prakash BS, Varma K, Michau D, Maglione M (2008). Deposition and dielectric properties of CaCu_3_Ti_4_O_12_ thin films deposited on Pt/Ti/SiO_2_/Si substrates using radio frequency magnetron sputtering. Thin Solid Films.

[98] Nigro RL, Toro RG, Malandrino G, Fragalà IL, Fiorenza P, Raineri V (2007). Effects of high temperature annealing on MOCVD grown CaCu_3_Ti_4_O_12_ films on LaAlO_3_ substrates. Surf. Coat. Technol..

[99] Nigro RL, Toro RG, Malandrino G, Fragalà IL, Fiorenza P, Raineri V (2007). Chemical stability of CaCu_3_Ti_4_O_12_ thin films grown by MOCVD on different substrates. Thin Solid Films.

[100] Jin S, Xia H, Zhang Y, Guo J, Xu J (2007). Synthesis of CaCu_3_Ti_4_O_12_ ceramic via a sol–gel method. Mater. Lett..

[101] Feng L, Wang Y, Yan Y, Cao G, Jiao Z (2006). Growth of highly-oriented CaCu_3_Ti_4_O_12_ thin films on SrTiO_3_ (1 0 0) substrates by a chemical solution route. Appl. Surf. Sci..

[102] Maurya D, Singh DP, Agrawal DC, Mohapatra YN (2008). Preparation of high dielectric constant thin films of CaCu_3_Ti_4_O_12_ by sol–gel. Bull. Mater. Sci..

[103] Smith M, Turner AF (1965). Vacuum deposited thin films using a ruby laser. Appl. Opt..

[104] Fang L, Shen MR, Li ZY (2006). Effect of double-sided CaTiO_3_ buffer layers on the electrical properties of CaCu_3_Ti_4_O_12_ films on Pt/Ti/SiO_2_/Si substrates. J. Appl. Phys..

[105] Fang L, Shen M, Yang J, Li Z (2006). Reduced dielectric loss and leakage current in CaCu_3_Ti_4_O_12_ /SiO_2_/ CaCu_3_Ti_4_O_12_ multilayered films. Solid State Commun..

[106] Fiorenza P, Nigro RL, Sciuto A, Delugas P, Raineri V, Toro RG, Catalano MR, Malandrino G (2009). Perovskite CaCu_3_Ti_4_O_12_ thin films for capacitive applications: from the growth to the nanoscopic imaging of the permittivity. J. Appl. Phys..

[107] Jiménez R, Calzada ML, Bretos I, Goes JC, Sombra ASB (2007). Dielectric properties of sol–gel derived CaCu_3_Ti_4_O_12_ thin films onto Pt/TiO_2_/Si(1 0 0) substrates. J. Eur. Ceram. Soc..

[108] Shen YS, Chiou BS, Ho CC (2008). Effects of annealing temperature on the resistance switching behavior of CaCu_3_Ti_4_O_12_ films. Thin Solid Films.

[109] Li YW, Hu ZG, Sun JL, Meng XJ, Chu JH (2008). Preparation and properties of CaCu_3_Ti_4_O_12_ thin film grown on LaNiO_3_-coated silicon by sol–gel process. J. Cryst. Growth.

[110] Li YW, Shen YD, Hu ZG, Yue FY, Chu JH (2009). Effect of thickness on the dielectric property and nonlinear current-voltage behavior of CaCu_3_Ti_4_O_12_ thin films. Phys. Lett. A.

[111] Brattain WH, Bardeen J (1953). Surface properties of germanium. Bell Syst. Tech. J..

[112] Seiyama T, Kato A, Fujiishi K, Nagatani M (1962). A new detector for gaseous components using semiconductive thin films. Anal. Chem..

[113] Kanazawa E, Sakai G, Shimanoe K, Kanmura Y, Teraoka Y, Miura N, Yamazoe N (2001). Metal oxideMetal oxide semiconductor N_2_O sensor for medical use. Sens. Actuators B: Chem..

[114] Moseley PT (1997). Solid state gas sensors. Meas. Sci. Technol..

[115] Sekimoto S, Nakagawa H, Okazaki S, Fukuda K, Asakura S, Shigemori T, Takahashi SA (2000). Fibre-optic evanescent-wave hydrogen gas sensor using palladium-supported tungsten oxide. Sens. Actuators B: Chem..

[116] Morazzoni F, Scotti R, Origoni L, Arienzo MD, Jimenez I, Cornet A, Morante JR (2006). Mechanism of NH_3_ interaction with transition metal-added nanosized WO_3_ for gas sensing: in situ electron paramagnetic resonance study. Catal. Today.

[117] Albert KJ, Lewis NS, Schauer CL, Sotzing GA, Stilzel SE, Vaid TP, Walt DR (2000). Cross-reactive chemical sensor arrays. Chem. Rev..

[118] Shimizu Y, Egashira M (1999). Basic aspects and challenges of semiconductor gas sensors. MRS Bull..

[119] Martinelli G, Carotta MC, Traversa E, Ghiotti G (1999). Thick-film gas sensors based on nanosized semiconducting oxide powders. MRS Bull..

[120] Tomchenko AA, Harmer GP, Marquis BT (2005). Detection of chemical warfare agents using nanostructured metal oxide sensors. Sens. Actuators B: Chem..

[121] Tomchenko AA, Harmer GP, Marquis BT, Allen JW (2003). Semiconducting metal oxide sensor array for the selective detection of combustion gases. Sens. Actuators B: Chem..

[122] Franke ME, Koplin TJ, Simon U (2006). Metal and metal oxide nanoparticles in chemiresistors: does the nanoscale matter. Small.

[123] Yamazoe N (1991). New approaches for improving semiconductor gas sensors. Sens. Actuators B: Chem..

[124] Macholdt HT, Vaneldik R (1985). High pressure effects on ligand substitution reactions of molybdenum(0) carbonyl complexes. Transit. Met. Chem..

[125] Magner G, Savy M, Scarbeck G, Riga J, Verbist JJ (1981). Effects of substitution of iron by molybdenum in the naphthalocyanine structures upon their electrocatalytic properties for O2 reduction and evolution in alkaline media. J. Electrochem. Soc..

[126] Simon I, Bârsan N, Bauer M, Weimar U (2001). Micromachined metal oxide gas sensors: opportunities to improve sensor performance. Sens. Actuators B: Chem..

[127] Li M, Chen XL, Zhang DF, Wang WY, Wang WJ (2010). Humidity sensitive properties of pure and Mg-doped CaCu_3_Ti_4_O_12_. Sens. Actuators B: Chem..

[128] Miao LJ, Xin JW, Shen ZY, Zhang YJ, Wang HY, Wub AG (2013). Exploring a new rapid colorimetric detection method of Cu^2+^ with high sensitivity and selectivity. Sens. Actuators B: Chem..

[129] Stankova M, Vilanova X, Calderer J, Llobet E, Brezmes J, Gracia I, Cane C, Correig X (2006). Sensitivity and selectivity improvement of rf sputtered WO_3_ microhotplate gas sensors. Sens. Actuators B: Chem..

[130] Yin XT, Guo XM (2014). Selectivity and sensitivity of Pd-loaded and Fe-doped SnO_2_ sensor for CO detection. Sens. Actuators B: Chem..

[131] Yang S, Liu Y, Chen W, Jin W, Zhou J, Zhang H, Zakharova GS (2016). High sensitivity and good selectivity of ultralong MoO_3_ nanobelts for trimethylamine gas. Sens. Actuators B: Chem..

[132] Wang M, Zhu L, Zhang C, Gai G, Ji X, Li B, Yao Y (2016). Lanthanum oxide@ antimony-doped tin oxide with high gas sensitivity and selectivity towards ethanol vapor. Sens. Actuators B: Chem..

[133] Chung SY, Kim ILD, Kang SJL (2004). Strong nonlinear current-voltage behaviour in perovskite-derivative calcium copper titanate. Nat. Mater..

[134] Parra R, Savu R, Ramajo LA, Ponce MA, Varela JA, Castro MS, Bueno PR, Joanni E (2010). Sol–gel synthesis of mesoporous CaCu_3_Ti_4_O_12_ thin films and their gas sensing response. J. Solid State Chem..

[135] Zampetti E, Pantalei S, Pecora A, Valletta A, Maiolo L, Minotti A, Macagnano A, Fortunato G, Bearzotti A (2009). Design and optimization of an ultra thin flexible capacitive humidity sensor. Sens. Actuators B: Chem..

[136] Kim J, Yong K (2011). Mechanism Study of ZnO Nanorod-Bundle Sensors for H_2_S Gas Sensing. J. Phys. Chem..

[137] Mohammadzadeh F, Jahanshahi M, Rashidi AM (2012). Preparation of nanosensors based on organic functionalized MWCNT for H_2_S detection. Appl. Surf. Sci..

[138] Nisha R, Madhusoodanan KN, Vimalkumar TV, Vijayakumar KP (2015). Gas sensing application of nanocrystalline zinc oxide thin films prepared by spray pyrolysis. Bull. Mater. Sci..

